# Research Progress on Histone Modification Regulation Mechanisms and Breeding Applications in Plant Abiotic Stress Responses

**DOI:** 10.3390/plants15131955

**Published:** 2026-06-25

**Authors:** Yan-Shuang Liu, Nian Liu, Xu-Zhe Cui, Li-Na Liu, Ming-Yuan Zhang, Hui-Chun Wang

**Affiliations:** 1 College of Life Sciences, Qinghai Normal University, Xining 810008, China; 15004215046@163.com (Y.-S.L.);; 2 Qilian Mountain Southern Slope Forest Ecosystem Research Station, Huzhu 810500, China; 3 Academy of Plateau Science and Sustainability, Qinghai Normal University, Xining 810008, China; 4 Key Laboratory of Medicinal Animal and Plant Resources on the Qinghai-Tibet Plateau, Xining 810099, China

**Keywords:** plants, abiotic stress, histone modification, epigenetics, stress signaling, transgenerational inheritance, epigenetic editing

## Abstract

Abiotic stresses severely restrict plant growth, development, and crop yield. Histone modification functions as a key epigenetic regulator in plant stress adaptation. This review systematically summarizes the major types of histone modifications (e.g., acetylation, methylation) and their catalytic enzyme systems. It clarifies the regulatory patterns of chromatin remodeling and gene expression under diverse abiotic stress conditions, like extreme temperature changes, persistent drought, elevated salinity, and heavy metal exposure, and reveals the crosstalk networks between histone modifications and ABA, CBF/DREB, and ROS signaling pathways. It also discusses the transgenerational inheritance of stress-induced histone modification variations and their molecular basis, and introduces the application of CRISPR/Cas9 and dCas9-based epigenetic editing in improving crop stress resistance. Currently, research on histone modification in plateau crops remains fragmented: studies mostly focus on single stress rather than combined multiple abiotic stresses, lack tissue-specific epigenetic regulatory maps for native plateau plants, and the field application of epigenetic breeding technologies is seriously insufficient. Considering the compound stresses, including low temperature, drought, salinization, and heavy metals, on the Qinghai–Tibet Plateau, this review identifies current research gaps, such as tissue specificity, multi-stress crosstalk, and field application, and proposes future directions, including multi-omics analysis, stress adaptation mechanisms of plateau plants, and precise epigenetic breeding. Overall, this review fills the research gap of systematic collation on histone-mediated stress tolerance epigenetics under plateau combined abiotic stresses, and provides a theoretical reference for epigenetic research on plant stress resistance and for the improvement of plateau crops.

## 1. Introduction

As sessile organisms, plants are persistently exposed to a complex environment of multiple abiotic stresses, including drought, salinity, extreme temperatures, and heavy metals [1]. The Qinghai–Tibet Plateau is a critical ecological security barrier and a distinctive agricultural and pastoral production base in China. To adapt to harsh environmental stresses, plants have progressively developed complex molecular regulatory networks. Among these, epigenetic regulation, which accurately regulates gene transcription without modifying the DNA sequence, has become a crucial mechanism allowing plants to quickly sense and adapt to abiotic stresses [2,3].

Epigenetic variations are mainly mediated through DNA methylation, histone modification, non-coding RNA (ncRNA) regulation, and chromatin remodeling. As the most dynamic and specific mode of epigenetic regulation, histone modification influences gene accessibility and transcriptional activity by altering chromatin structure [4].

This review focuses on the full-chain regulatory network of histone modification, covering enzyme regulation, stress response, signaling crosstalk, transgenerational inheritance, and engineering applications. It systematically analyzes the regulatory patterns and molecular mechanisms of core modification types (acetylation, methylation, and others) under diverse abiotic stresses, illustrates the crosstalk between histone modifications and plant stress signaling pathways, and dissects the regulatory basis of stress-induced transgenerational inheritance of histone modifications. Finally, it summarizes existing research limitations and prospects future directions, aiming to provide a systematic overview for epigenetic regulation studies on plant abiotic stress responses.

Nevertheless, existing global studies on plant histone modification mainly focus on conventional crops grown in plain areas and single stress treatments. Three major research gaps exist in plateau crop epigenetics: (1) Most studies ignore the synergistic regulation mechanism of histone modifications under the co-occurrence of low temperature, drought, salinity and heavy metals; (2) The tissue-specific dynamic changes and regulatory differences of histone marks in plateau endemic crops are rarely reported; (3) The transgenerational epigenetic memory and field application of epigenetic editing technologies for plateau crops remain poorly explored.

## 2. Plant Histone Modifications

### 2.1. Major Types of Plant Histone Modifications

Histones are the basic structural units of eukaryotic chromatin, which form nucleosomes by wrapping DNA [5]. These modified histone marks are specifically recognized and bound by reader proteins, which, together with histone writers and erasers, orchestrate chromatin function and gene expression. Distinct reader domains specifically recognize typical activating and repressive histone marks like *H3K4me3* and *H3K27me3*, thereby modulating the expression of stress-related genes in plants. Within the regulatory networks underlying plant abiotic stress responses, histone acetylation and methylation represent the most systematically investigated and functionally critical modification types, providing the core molecular basis for epigenetic regulation of plant stress responses.

Histone acetylation is mediated by histone acetyltransferases (HATs). These enzymes attach acetyl groups to lysine sites on histones. In contrast, histone deacetylases (HDACs) are responsible for removing acetyl groups, promoting chromatin condensation and thereby mediating transcriptional repression [6,7]. Studies have shown that the expression of numerous stress-responsive genes is dynamically regulated by histone acetylation status during plant abiotic stress responses [8]. For instance, rice *RPD3/HDA1*-type HDAC *OsHDA716* negatively modulates plant drought resistance [9], and its dual regulatory mechanism is described in detail in the following section. Histone methylation is dynamically controlled by histone methyltransferases (HMTs) and histone demethylases (HDMs) [10,11]. Unlike acetylation, its regulatory effect depends on modification sites and methylation levels: *H3K4me3* acts as a transcriptional activation mark, while *H3K27me3* mainly mediates gene silencing [12,13]. Under abiotic stresses, the dynamic balance of methylation at specific histone sites modulates the expression of stress-related genes and plant stress adaptation [14].

Ubiquitination and SUMOylation are two vital ubiquitin-like modification systems. Beyond the common phosphorylation sites such as *H3S10* and *H3S28*, *H3T3ph* and *H2AX* phosphorylation (*γ-H2AX*) are representative histone phosphorylation marks in plants, with distinct functions under abiotic stress. *H3T3ph* is mainly linked to cell cycle progression. Catalyzed by HASPIN kinases, its abundance correlates with mitotic cell proportion [15]. Salt stress induces G2/M arrest and microtubule damage, reducing the overall level and disrupting the distribution of *H3T3ph*. Heat stress impairs chromosome segregation, leading to fluctuating *H3T3ph* signals; currently, no evidence proves its direct regulation of stress gene transcription [16,17,18]. As a classic DNA damage marker, *γ-H2AX* is strongly induced by heat and salt stress. Severe heat stress causes massive DNA breaks and a sharp elevation of *γ-H2AX*, while salt stress triggers moderate *γ-H2AX* accumulation due to oxidative damage. *γ-H2AX* facilitates DNA repair and enhances plant stress resistance. Collectively, heat and salinity shape obviously different histone phosphorylation landscapes. Heat stress mainly triggers DNA damage-associated *γ-H2AX* accumulation and disordered distribution of cell cycle-related *H3T3ph*, while salt stress leads to a global decrease of *H3T3ph* and mild upregulation of *γ-H2AX*. Such divergent phosphorylation patterns constitute an important epigenetic distinction between these two abiotic stresses.

SUMOylation can rapidly respond to heat shock, drought, cold, and salt stresses, enhancing plant tolerance by modulating target protein trafficking, transcriptional regulation, and apoptotic pathways. Profound and dynamic crosstalk exists between SUMOylation and histone acetylation pathways, which dominate chromatin remodeling and plant abiotic stress responses [19,20]. SUMOylation modulates the activity of histone acetyltransferases (HATs) and histone deacetylases (HDACs), and further precisely controls chromatin accessibility and gene transcription [20]. As a core SUMO E3 ligase, *SIZ1* physically interacts with HDAC HDA6 and negatively regulates its deacetylation activity [21]. This modification maintains histone acetylation levels at target gene promoters and facilitates gene expression, enabling SUMO to bidirectionally regulate developmental and stress signals via fine-tuning HDAC functions [21].

Under heat stress, genome-wide redistribution of SUMO on chromatin drives the transition from plant growth to stress adaptation [22]. SUMO enrichment at gene promoters activates heat-responsive genes, including *DREB2A* and *HSP* family genes, and stabilizes key transcription factors to enhance thermotolerance [22,23]. In salt stress, SUMOylation also improves salt tolerance by maintaining the stability of stress-related transcription factors and activating downstream defense pathways [24]. SUMOylation and histone acetylation function in both synergistic and antagonistic manners. SUMO represses HDAC activity to indirectly promote histone acetylation, and also modulates HAT activity to activate stress-related genes directly [20,21]. Collectively, the crosstalk between SUMOylation and HAT/HDAC pathways constructs a flexible epigenetic regulatory network for plant stress adaptation [25,26].

Ubiquitination relies on two main chain types: K48-linked chains mediate protein degradation, and K63-linked chains participate in DNA repair and signal assembly. In Arabidopsis, PUB25/26 catalyze K48 ubiquitination of *ICE1* to regulate cold response [27].

Histone variants, lactylation, and crotonylation represent three key epigenetic mechanisms that play central roles in sensing environmental signals, integrating metabolic status, and driving transcriptional reprogramming. Studies have confirmed that specific histone variants such as *H3.14* and *H2A.Z* can directly respond to stress signals and modulate the accessibility of stress-related genes by altering nucleosome stability [28,29]. Meanwhile, modifications derived from the metabolic byproducts lactate and crotonate—namely lactylation and crotonylation—directly couple cellular metabolism to chromatin function, providing a sophisticated mechanism that coordinates energy status with gene expression for plant survival under stress [30,31].

Histone lactylation is a novel post-translational modification that directly links glycolytic metabolism to gene regulation. This modification is predominantly induced by hypoxia and altered cellular metabolic status rather than general abiotic stresses. Stress-triggered lactate accumulation exhibits distinct tissue and organ specificity in plants [32,33]. Plant roots are the primary site of lactate production: under waterlogging and hypoxic conditions, root cells switch to anaerobic glycolysis, and lactate dehydrogenase (LDH) catalyzes the conversion of pyruvate to lactate to sustain energy metabolism [32,33]. By contrast, above-ground tissues such as leaves mainly rely on aerobic respiration. Under drought and salinity stresses, plants tend to synthesize compatible solutes instead of accumulating lactate for osmotic adjustment, so massive lactate fermentation rarely occurs in aerial organs [34,35,36]. Different LDH isoforms further lead to functional differentiation across tissues [37]. To date, it remains an open question whether stress-induced lactate accumulation and subsequent histone lactylation occur ubiquitously in all plant tissues or are restricted to root-specific organs, which is a key issue to be addressed in future research. In maize roots, drought stress induces lactate accumulation, which leads to a significant increase in the lactylation of proteins, especially histones. Profiling of histone lactylation reveals substantial differences in modification patterns between drought-tolerant and drought-sensitive varieties, and this modification is positively correlated with drought tolerance. As a typical metabolism–epigenetics coupling mechanism, histone lactylation (Kla) constructs a complete regulatory cascade linking glycolytic metabolism to stress-responsive gene expression [38,39]. Under drought, waterlogging, and hypoxic conditions, plant aerobic respiration is inhibited, and glycolysis becomes the major pathway for ATP production. Pyruvate generated from glycolysis is converted to L-lactate, catalyzed by LDH [39,40]. Lactate cannot modify histones directly; it is activated to lactyl-CoA by acyl-CoA synthetase short-chain family member 2 (ACSS2) [41,42]. ACSS2 is distributed in both cytoplasm and nucleus, and nuclear-localized ACSS2 provides sufficient lactyl-CoA around chromatin, laying a material foundation for histone lactylation [41,42,43]. Drought can transcriptionally upregulate LDH and ACSS2 in maize roots and drive local accumulation of lactyl-CoA [44].

The dynamic balance of histone lactylation is governed by writers and erasers. The *p300/CBP* complex, a member of the histone acetyltransferase (HAT) superfamily, acts as the primary lactyltransferase to catalyze histone lactylation [40,41]. Lactylation competes with acetylation for the same lysine residues, such as H3K9 and *H3K14*; the fluctuation of intracellular lactyl-CoA concentration alters the substrate preference of *p300/CBP* and switches chromatin status [41,45]. Class I HDACs (HDAC1-3) and sirtuin family proteins (SIRT1, SIRT2) function as delactylases to remove lactyl groups and maintain modification homeostasis [46,47,48,49].

Bromodomain and YEATS domains serve as core reader modules to recognize lactylation marks [41,50]. After binding to modified histones, these proteins recruit transcription complexes and chromatin remodelers to regulate downstream gene transcription. In maize, histone lactylation is enriched at the promoter and transcription start sites of drought-responsive genes, relaxing chromatin structure. It further activates ABA signaling, ROS scavenging, and osmotic regulation-related genes such as NCED, *SnRK2*, SOD, and CAT, thereby enhancing plant drought resistance [44,51]. The tissue-specific distribution of LDH and ACSS2 also explains the distinct lactylation levels between underground and above-ground tissues in plants [44]. Histone crotonylation is closely associated with unsaturated fatty acid metabolism [30]. Different from lactylation, crotonylation originates from plant lipid metabolism [52,53]. Salt and heat stress accelerate the degradation of unsaturated fatty acids to generate crotonate. ACSS2 is involved in crotonyl-CoA synthesis, though its catalytic efficiency is limited, suggesting alternative metabolic pathways exist [54]. Crotonylation shares *p300/CBP* and *HDAC/SIRT* enzymes with other acyl modifications and competes for histone lysine sites [55,56]. YEATS domains are the dominant readers for crotonylation [57]. This modification strongly activates promoters and enhancers, and upregulates *SOS*, *NHX*, and *HSP* genes in wheat to enhance salt and thermotolerance [58,59].

As a donor, the intracellular concentration of crotonyl-CoA is tightly regulated by metabolic status. Research indicates that crotonylation is commonly enriched at active enhancers and promoters and exhibits stronger transcriptional activation capacity than acetylation [60,61]. During plant stress responses, reprogramming of lipid metabolism is a common physiological adaptation. Crotonylation may act as a sensor that converts lipid metabolic signals into changes in chromatin structure, thereby activating specific stress defense genes. Histone variants mediate plant abiotic stress responses through chromatin remodeling [62,63]. ROS and MAPK signals activate *SWI/SNF* and *INO80* complexes to replace canonical histones with *H2A.Z* and *H3.14* [64,65]. Structural differences of these variants change nucleosome stability and chromatin accessibility [28,66]. Dynamic redistribution of *H2A.Z* at gene promoters initiates gene transcription [67,68]. These variants recruit transcriptional regulators and activate SOS and CBF pathways to confer salt and cold tolerance [18,69,70]. The *TORC1*-*Rpd3* pathway also crosstalks with *H2A.Z* to coordinate stress and metabolism [71,72].

As essential acyl donors, lactyl-CoA and crotonyl-CoA are regulated by plant glycolipid and energy metabolism pathways. However, the crosstalk between glycolipid metabolism and acyl-CoA homeostasis under adverse conditions remains poorly understood. Existing omics data reveal stress-specific distribution features of the two modifications: Histone lactylation is mainly enriched near gene transcription start sites in drought-stressed maize [30], while salt stress significantly remodels crotonylation profiles in wheat chloroplast-related proteins. Nevertheless, systematic genome-wide comparison of their distribution landscapes across heat, cold, salt, and heavy metal stresses has not been reported. All three acylation types (lactylation, crotonylation, and acetylation) occupy overlapping lysine residues on histones [60,61]. Although this competitive regulatory mode has been validated in animals and partial plant models, how stress signals dynamically tune this crosstalk to regulate gene expression is still an open question. Clarifying these three puzzles will greatly advance our understanding of metabolism-coupled epigenetic regulation in plant stress adaptation. Notably, excessive or ectopic histone modifications may also exert deleterious effects on plant growth and development. Plants often face physiological trade-offs between stress tolerance and normal growth when maintaining stress-related histone modification patterns. Currently, there are also inconsistent findings regarding the tissue specificity and stress preference of lactylation and crotonylation, and the shared substrate competition mechanism among acylation modifications remains a major unresolved issue in this field.

### 2.2. Regulatory Enzymes of Histone Modifications

The histone acetyltransferase *Hat1*, a member of the HAT family, transfers acetyl groups to the ε-amino groups of lysine residues on histones H4 and, to a lesser extent, H3 [73]. This type of epigenetic modification diminishes the positive charge of lysine and lessens electrostatic binding between histones and negatively charged DNA strands [74], rendering chromatin in a relaxed euchromatin state. This provides accessible binding sites for transcription factors and RNA polymerases, thus triggering widespread gene transcription. The plant HAT family mainly comprises the GNAT, MYST, and *p300/CBP* subfamilies [75]. Although the substrate specificities of different subfamilies partially overlap, each favors preferred histone lysine sites, including *H3K9*, *H3K14*, *H3K27*, *H4K5*, *H4K8*, *H4K12*, and *H4K16* [76]. Such site specificity provides the molecular basis for the precise regulation of histone acetylation. Notably, different HAT subfamilies display obvious spatiotemporal divergence upon drought and heat exposure. The GNAT subfamily is predominantly activated at the early stage in roots and leaves under drought [77,78], facilitating acetylation of stress-related genes [79]. The MYST subfamily, by contrast, functions mainly under sustained heat stress and is highly expressed in aboveground tissues [80,81]. The *p300/CBP* subfamily responds to both stresses universally and acts as a shared component in diverse stress signaling [82]. Such subtype-specific spatiotemporal responses enable plants to assemble dedicated epigenetic networks for different abiotic stresses [82].

Opposite to HATs, HDACs are responsible for removing acetyl groups from lysine residues of histones, restoring the positive charge of lysines, promoting chromatin condensation into heterochromatin, and thereby mediating transcriptional repression [83]. Plant HDACs are classified into three major families based on sequence characteristics and functional differences: *RPD3/HDA1*, *SIR2*, and *HD2* [84]. Rice *OsHDA716*, a representative member of the *RPD3/HDA1* family, exhibits dual regulatory capabilities. Apart from modifying histones to remodel chromatin, it acts on the non-histone protein *OsbZIP46*. By cooperating with E3 ubiquitin ligase *OsPUB75*, *OsHDA716* triggers the deacetylation and degradation of *OsbZIP46*, thereby suppressing rice drought tolerance. This indicates that HDACs can regulate stress signaling via both chromatin modification and modulation of pathway-related proteins [9,85].

Histone modification enzymes exhibit remarkable species-specific distribution and functional divergence in plants, which are closely associated with species characteristics and stress adaptation [84]. As observed in typical model plants, *RPD3/HDA1*-type HDACs display distinct functional priorities between monocot and dicot plants. In monocot crops such as rice and maize, these enzymes mainly function through the modification and regulation of non-histone substrates, mediating stress responses by modulating the activity of key proteins in signaling pathways [9,85]. In dicot plants such as Arabidopsis, their primary role is histone modification, influencing the transcription of stress-responsive genes by regulating chromatin structure [86,87]. The GNAT subfamily of HATs shows significantly higher expression in stress-tolerant germplasm than in conventional crops. Its elevated expression enhances adaptation to drought, salinity, and other stresses by increasing acetylation of stress-responsive genes. For example, 30 TaHAT genes have been identified in wheat, among which GNAT subfamily members participate in plant growth, development, and stress responses [77]. In pepper, HAT genes are differentially expressed during fruit development and ripening, indicating their roles in regulating secondary metabolite biosynthesis. In perennial woody plants such as grapes, SIR2-type HDACs are highly expressed [88]. HD2-type HDACs are plant-specific and show distinct expansion in monocots [89]. This distribution pattern provides an enzymatic basis for dissecting the stress adaptation mechanisms of diverse plant species. However, this functional divergence between monocots and dicots is summarized from limited cases. Meanwhile, how HDACs dynamically switch between histone and non-histone substrates under different stresses is still poorly understood and needs further exploration.

Of note, the substrate selectivity of histone-modifying enzymes has emerged as a key research frontier in plant epigenetics [8]. Current studies have confirmed that enzymes such as HDACs can target both chromatin-bound histone substrates and cytoplasmic/nuclear non-histone signaling proteins [85,90]. However, how these enzymes dynamically adjust their catalytic preferences and partition functional targets when plants are exposed to different types of abiotic stresses remains largely uncharacterized [91]. Clarifying this dynamic targeting mechanism will help fully reveal the dual regulatory modes of histone-modifying enzymes in plant stress responses.

### 2.3. Synergistic and Antagonistic Regulation of Histone Modifications

Histone acetylation and methylation exhibit a striking functional antagonism, and their dynamic balance constitutes the core of regulating chromatin status and gene expression. Acetylation counteracts the positive charges on histone lysine residues, weakening electrostatic attraction between histones and DNA. This facilitates the transition of chromatin from condensed heterochromatin to loose euchromatin, which enhances the accessibility of transcriptional machinery and is closely linked to gene activation [92]. In comparison, histone methylation fails to change the charge characteristics of amino acids, and its regulatory effects are closely determined by methylation locations and degrees. For instance, H3 lysine 4 trimethylation (*H3K4me3*) commonly acts as an activating epigenetic hallmark, while *H3K27me3* and H3K9me3 are well-established repressive chromatin modifications [93,94]. *H3K27me3* specifically recruits the Polycomb repressive complex 2 (*PRC2*) to chromatin regions, maintaining chromatin condensation and mediating gene silencing [95]. At the promoter regions of many stress-responsive genes, the deposition of *H3K27me3* is often accompanied by reduced acetylation of H3/H4. Together, they establish a stable transcriptionally repressive environment, allowing plants to maintain low-level stress responses under normal growth conditions and avoid unnecessary energy consumption. Studies have shown that the rice SLENDER RICE1 (*SLR1*) protein forms a ternary complex with *PRC2* and HDACs to mediate gene silencing via *H3K27me3* [96]. Under cold stress conditions, potato plants exhibit elevated chromatin openness and bivalent histone marks of *H3K4me3* and *H3K27me3* at active gene loci [94], indicating that genes are maintained in a “poised state” that is neither fully activated nor deeply repressed, enabling rapid responses upon receiving specific signals.

When plants perceive stress signals, histone demethylases (such as JmjC family members) can remove repressive marks, including *H3K27me3*, thereby releasing gene silencing [92,97]. Meanwhile, histone acetyltransferases (HATs) catalyze acetylation at sites including *H3K9* and *H3K14*, further loosening chromatin structure [92,98]. Spatially and temporally, the removal of repressive methylation marks always precedes histone acetylation; different modification sites can recognize and cooperate with each other via reader proteins. Under prolonged stress, chromatin will dynamically switch between active and poised states to balance defense and growth. This coordinated “de-repression–activation” cascade rapidly triggers the transcription of downstream defense genes and mediates plant stress responses [30,91]. For example, in *Arabidopsis*, jasmonate phytohormones and histone deacetylase 6 (*HDA6*) promote global histone acetylation and methylation modifications at the initial stage of acute stress adaptation [98].

Plants employ epigenetic modifications such as histone acetylation and methylation to precisely respond to abiotic stresses, including cold and drought. These modifications act both synergistically and antagonistically to collectively ensure plant adaptability to environmental changes. In the cold stress response, the CBF/DREB transcription factor family serves as the central regulatory hub [99]. Under normal growth conditions, CBF loci are typically silenced by the repressive mark *H3K27me3*, maintaining low expression levels [13]. Upon sensing low temperatures, plants induce histone demethylases that remove the repressive *H3K27me3* mark from CBF loci, facilitating their transcriptional activation [13]. Concurrently, HAT activity is enhanced, depositing the activating marks *H3K4me3* and H3/H4 acetylation at the same regions [100,101]. The coordinated action of these multiple activating modifications promotes chromatin opening, enabling rapid and robust induction of CBF genes, which in turn activate the entire cold-responsive gene network and enhance plant chilling tolerance [13,102]. Similarly, in rice, *OsHDA716* negatively modulates drought responses. It not only represses stress-related genes via histone deacetylation, but also targets the ABA pathway component *OsbZIP46*, linking chromatin regulation with protein post-translational modification networks [9,85].

In addition to the synergy and antagonism between acetylation and methylation, histone phosphorylation can trigger a cascade reaction with acetylation and methylation. Acting as a “priming” modification, it facilitates the recruitment of subsequent modifying enzymes. This regulatory mode shows remarkable stress specificity in response to abiotic stresses such as salt stress and heat stress, further diversifying the regulatory network of histone modifications [103]. In the heat stress response, *H3S10ph* in *Arabidopsis thaliana* mediates the deposition of *H3K4me3* at heat shock protein (*HSP*) gene loci [104]. As a “priming” mark, *H3S10ph* provides binding sites for the recruitment of HMTs, which in turn promote robust expression of HSP genes through H3K4me3-dependent activation, thereby enhancing thermotolerance [104]. Furthermore, in maize under drought stress, histone modifications such as *H3K9ac* are dynamically modulated at retrotransposon loci, accompanying chromatin compaction [105]. In drought stress, histone modifications including *H3T3ph* and *H3K9ac* cooperatively activate the expression of osmoprotectant biosynthesis genes, underpinning plant drought adaptation [105,106]. This cascade regulatory mechanism, relying on mutual recognition and recruitment among modifications, enables plant cells to mount highly specific and amplified transcriptional responses to weak environmental stress signals, representing a vital epigenetic strategy for precise plant adaptation to adverse environments [107]. Although the synergistic and antagonistic relationships between histone modifications have been widely recognized, the exact spatiotemporal order of modification turnover and the fine regulatory mechanism of chromatin state switching under long-term stress still lack unified conclusions.

## 3. Regulatory Roles of Histone Modification in Plant Abiotic Stress Responses

As revealed in the previous section, histone acetylation, methylation, and phosphorylation function together via cascade reactions, synergism, and antagonism to construct sophisticated epigenetic regulatory networks. These molecular mechanisms enable plants to perceive and respond to environmental signals accurately. Drought, salinity, extreme temperatures, and heavy metal pollution are major abiotic stresses that threaten crop production and global food security. Herein, we systematically discuss the dynamic changes and functional performances of histone modifications when plants suffer from each type of abiotic stress.

### 3.1. Histone Modification Variations Under Extreme Temperatures

The Qinghai–Tibet Plateau is characterized by high altitude and large diurnal temperature differences, where plants are continuously exposed to persistent low temperatures and drastic temperature fluctuations. Histone modifications play a pivotal role in plant cold adaptation [108]. Extreme temperatures severely impair plant growth and development, and histone modifications participate in regulating the expression of heat shock protein (HSP) genes and cold-responsive genes. Under heat stress, increased HAT activity elevates the acetylation level at the promoters of HSP genes, thereby enhancing HSP expression and protecting cells from heat damage [109]. Under cold stress conditions, the C-repeat binding factor/dehydration-responsive element binding protein (CBF/DREB) family acts as the central regulatory hub. The activation of these transcription factors and the expression of their downstream cold-responsive (COR) genes are precisely modulated by histone methylation. For instance, *H3K4me3* facilitates the transcriptional activation of CBF and COR genes, while *H3K27me3* undergoes dynamic redistribution to fine-tune cold-specific gene expression [13].

To further clarify cold-responsive regulatory patterns, we systematically summarize the temporal dynamics of histone marks during short-term cold shock and long-term cold acclimation.

Plant cold adaptation is divided into two key stages. Short-term cold shock refers to exposure to non-lethal low temperature (e.g., 4 °C) within hours, when plants launch rapid emergency responses to sustain cellular homeostasis. Long-term cold acclimation lasts for days to weeks; plants remodel the transcriptome to accumulate antifreeze substances and acquire stable freezing tolerance [13,110]. Accumulated evidence proves that *H3K4me3*, *H3K27me3*, and histone acetylation act synergistically to regulate the CBF/DREB pathway throughout this process [94,111].

Short-term cold shock induces widespread chromatin remodeling and increased chromatin accessibility for transcription factor binding [94]. The antagonistic *H3K4me3* and *H3K27me3* undergo rapid genome-wide redistribution. Under normal conditions, COR genes are silenced by abundant *H3K27me3*. Upon cold exposure, *H3K27me3* declines sharply, and *H3K4me3* rises transiently, driving rapid activation of COR genes [13,112].

Besides single-mark dynamics, cold stress triggers bivalent *H3K4me3*-*H3K27me3* deposition on active genes in Arabidopsis and potato. This poised chromatin state enables flexible responses to temperature changes [94,111]. Histone acetylation further relaxes chromatin by neutralizing histone tail charges and facilitates the recruitment of transcriptional machineries [113].

During long-term cold acclimation, epigenetic regulation shifts from acute responses to steady-state maintenance and cold memory formation, with gene-specific histone changes. CBF expression peaks at the early cold stage and is then suppressed via negative feedback to avoid energy waste [114,115]. The *PRC2* complex mediates *H3K27me3* deposition at the CBF3 locus. The lncRNA SVALKA interacts with *PRC2* to inhibit CBF3 transcription, balancing cold resistance and normal growth [114]. Meanwhile, downstream COR genes accumulate stable *H3K4me3* to maintain antifreeze substance synthesis [116].

Long-term cold acclimation also establishes cold memory. After temperature recovery, pre-stressed plants retain stronger cold tolerance and faster stress responses, a trait linked to heritable histone patterns. Studies in cucumber reveal that *CsRBOH5.1* modulates ROS signaling to restore *H3K4me3* during recovery [117]. Cold-memory genes maintain high *H3K4me3*, whereas non-memory genes lose this mark. Knockout of *CsRBOH5.1* reduces global *H3K4me3* and eliminates cold memory [117,118]. Chromatin readers for bivalent marks also participate in *PRC2*-dependent cold memory [118].

Overall, histone modifications exhibit distinct temporal patterns during plant cold responses. Short-term cold shock causes rapid chromatin opening and *H3K4me3*/*H3K27me3* turnover to activate emergency genes. In long-term acclimation, *PRC2*-dependent *H3K27me3* represses upstream CBF genes, while *H3K4me3* sustains the activity of downstream COR genes. *H3K4me3* re-establishment during recovery forms the epigenetic basis of cold memory. Future studies should explore how histone writers and erasers sense temperature cues, and dissect crosstalk among different epigenetic layers, especially for cold-tolerant plants native to the Qinghai–Tibet Plateau [114,117].

### 3.2. Histone Modification Variations Under Drought Stress

The plateau region is characterized by arid conditions, low precipitation, and intense ultraviolet radiation, and the dynamic balance between histone acetylation and methylation directly governs plant drought tolerance. Drought stress is a major constraint on plant growth and crop yield, and plants can enhance their drought tolerance through histone modification regulation. For instance, the expression of numerous genes associated with the ABA signaling pathway (such as the *SnRK2* kinase family) and osmoprotectant biosynthesis genes is modulated by histone acetylation and methylation [3,119]. Accumulating evidence has demonstrated that H3K9 acetylation modulates the expression of key enzyme genes involved in flavonoid and ABA pathways, thereby improving drought resistance in sea buckthorn [120]. Conversely, histone deacetylases (HDACs) negatively regulate drought tolerance by reducing the acetylation levels of specific stress-responsive genes. A typical example is apple histone deacetylase 6 (*MdHDA6*), which suppresses drought tolerance by mediating deacetylation of stress-related genomic regions [121]. In addition, the R2R3-type transcription factor *SiMYB77* in sesame has been proven to enhance drought and salt tolerance in transgenic tobacco by maintaining stable osmotic metabolite accumulation and ROS homeostasis [122], and the expression of such transcription factors is frequently subjected to epigenetic regulation mediated by histone modifications.

### 3.3. Histone Modification Variations Under Salt Stress

The Qinghai–Tibet Plateau harbors extensive salinized grasslands, where plants frequently suffer from combined salinity and drought stress. Histone modifications act as crucial epigenetic regulators in maintaining ion homeostasis and osmotic adjustment. Salt stress disrupts cellular ion balance, triggers osmotic stress, and causes oxidative damage in plants. By modulating the expression of genes associated with reactive oxygen species (ROS) scavenging systems and stress-related processes, histone modifications substantially enhance plant salt tolerance [123,124]. For example, in castor bean, combined analysis of genomic transcription and histone methylation reveals that salt-induced changes in *H3K4me3* are closely associated with the expression of osmotic adjustment-related genes [123]. As a global hotspot of high-altitude salinized grasslands, the Qinghai–Tibet Plateau hosts the unique crop hulless barley (*Hordeum vulgare* L. var. nudum), which has evolved distinctive epigenetic regulatory mechanisms in response to salt stress. Basang et al. conducted genome-wide ChIP-seq analysis and found that the salt-tolerant hulless barley variety Z0119 and the salt-sensitive variety Z0226 exhibited opposite spatiotemporal changes in histone modifications *H3K4me3* (an activating mark) and *H3K27me3* (a repressive mark) under 200 mM salt stress. Twenty-four hours after stress, the number of genes with both modifications (*H3K4me3* + *H3K27me3*) was significantly upregulated (1.5-fold) in Z0119, and these genes were enriched in oxidative stress defense pathways, such as peroxisome activity and reactive oxygen species (ROS) scavenging. In contrast, the dual-modified genes in Z0226 were clustered in protein complex assembly processes, failing to effectively initiate stress resistance responses. Further studies revealed that the levels of *H3K4me3*/*H3K27me3* modifications at salt-tolerant orthologous genes in hulless barley (e.g., HVUL5H46981.2, an ortholog of rice OsZFP182) were highly coordinated with gene expression. By activating the antioxidant system and maintaining ion homeostasis, these genes confer high salt tolerance to hulless barley. This study, for the first time, revealed that the coordinated regulation of *H3K4me3* and *H3K27me3* serves as a key epigenetic basis for salt stress adaptation in plateau crops, providing a unique extreme habitat case for dissecting plant salt-tolerance epigenetic mechanisms [124]. Xu et al. (2022) further performed multi-omics integrative analysis and found that low-methylated regions (LMRs) in hulless barley under salt stress strongly overlapped with *H3K27me3*, enriched binding sites for salt-stress-related transcription factors such as TCP, MYB, and NAC, and synergistically regulated the expression of salt-tolerant genes together with *H3K4me3* [125]. This established an epigenetic regulatory network for the salt stress response in hulless barley, further refining the epigenetic mechanisms underlying salt tolerance in plateau crops [125]. Besides Tibetan hulless barley, oat (*Avena sativa*) and sea buckthorn (*Hippophae rhamnoides*) are dominant salt-tolerant crops on the Qinghai–Tibet Plateau. A large number of transcriptome and physiological datasets have been reported for salt-stressed oat [126,127]. Based on the conserved epigenetic mechanisms of gramineous plants, *H3K4me3* and *H3K27me3* dynamically regulate the transcription of salt-responsive genes such as *SOS1* and *NHX*, and histone acetylation modulated by HATs/HDACs also participates in salt adaptation [128,129]. For sea buckthorn, existing ChIP-seq data confirm that *H3K9ac* and bivalent *H3K4me3*-*H3K27me3* marks control stress-related gene expression and cooperate with RNA epigenetic modifications [124,130]. Comparatively, annual oat relies on rapid histone acetylation responses, while perennial sea buckthorn tends to form stable epigenetic regulation [131,132]. Nevertheless, high-resolution genome-wide epigenomic datasets of the two crops are still deficient, which limits in-depth multi-omics analysis [133].

As summarized above, hulless barley, oat, and sea buckthorn present conserved *H3K4me3/H3K27me3* regulatory patterns under salt stress, while distinct interspecific characteristics are also observed. Annual gramineous crops respond rapidly via histone modification, whereas perennial sea buckthorn establishes relatively stable epigenetic regulation. Current relevant studies are mostly based on scattered ChIP-seq data, and systematic analyses for plateau crops under combined stresses remain insufficient.

### 3.4. Histone Modification Variations Under Heavy Metal Stress

Heavy metal pollution acts as a key abiotic stressor that adversely affects plant growth, development, and crop yield. It exerts phytotoxic effects by inducing oxidative stress, disrupting cell membrane integrity, disturbing enzyme activities, and obstructing nutrient absorption. To cope with such adverse conditions, plants have evolved sophisticated molecular defense networks. As essential regulatory mechanisms that modulate gene expression without altering DNA sequences, epigenetic modifications are indispensable for plant adaptation to heavy metal stress [134]. Numerous recent studies have confirmed that permissive histone marks, including *H3K4me3* and histone acetylation, dominate the regulation of genes related to *PCS* production and cell wall structural modification [8].

As a canonical transcriptional activation mark, *H3K4me3* is typically enriched at the promoter regions of actively transcribed genes. Under heavy metal stress, plant cells enhance the activity of specific histone methyltransferases (HMTs), which deposit *H3K4me3* at the promoters of heavy metal-responsive genes, thereby recruiting the transcriptional machinery and facilitating chromatin relaxation. Phytochelatin synthase (*PCS*) is the key enzyme responsible for phytochelatin (PC) biosynthesis in plants. *PCs* efficiently chelate toxic heavy metal ions, such as cadmium (Cd), lead (Pb), and arsenic (As), in the cytoplasm to form weakly toxic or non-toxic complexes. These complexes are subsequently sequestered into vacuoles, thereby mitigating heavy metal cytotoxicity [135,136,137]. The establishment of *H3K4me3* relies on SET-domain family HMTs [138], whose activities are directly or indirectly modulated by heavy metal signaling. This cascade forms an adaptive regulatory axis consisting of stress perception, epigenetic reprogramming, and functional gene expression, enabling plants to cope with heavy metal toxicity.

Meanwhile, histone acetylation enhances plant tolerance and adaptation to heavy metals through the fine-tuning of gene expression [8]. This epigenetic modification relies on the dynamic equilibrium between histone acetyltransferases (HATs) and histone deacetylases (HDACs), which jointly determine chromatin accessibility and thereby modulate the transcription of heavy metal-responsive genes [139]. Under the induction of stress signals, HATs catalyze acetylation at specific lysine residues of histones H3 and H4, such as *H3K9*, *H3K14*, and *H4K5* [140]. Such acetylation neutralizes the positive charge of lysine residues, weakens the binding affinity between nucleosomes and DNA, loosens chromatin structure, facilitates the recruitment of transcription factors to gene promoters, and ultimately activates the transcription of stress-related genes. In addition to directly regulating chelation-related genes such as *PCS* to enhance heavy metal chelation capacity, histone acetylation also extensively modulates the expression of cell wall remodeling enzyme genes. The cell wall acts as the primary physical and chemical barrier against heavy metal invasion. Functional groups such as carboxyl and hydroxyl groups in major cell wall components, including pectin, hemicellulose, and xyloglucan, efficiently adsorb and immobilize heavy metal ions, restricting their intracellular migration and alleviating cytotoxicity [141]. Under heavy metal stress, plants dynamically remodel cell wall composition and structure, a process dependent on multiple modifying enzymes, such as pectin methylesterase (PME), expansins, and xyloglucan endotransglucosylase/hydrolases (*XTH*s) [142]. The expression of these cell wall modification genes is precisely controlled by histone acetylation status. Apart from histone acetylation, repressive histone mark *H3K27me3* also dynamically modulates the transcription of PME and *XTH*. Under normal growth conditions, *H3K27me3* accumulates at its promoter to maintain low basal expression, while heavy metal stress triggers the removal of *H3K27me3* to further activate gene expression [142]. Meanwhile, specific HATs are activated to deposit acetylation marks in the regulatory regions of these genes, stimulating their transcription and strengthening cell wall immobilization capacity and barrier function. For instance, the *XTH* gene family contributes critically to metal ion resistance by mediating cell wall remodeling. Heavy metal ATPase (HMA) transporters are key functional proteins responsible for vacuolar compartmentalization of heavy metals. The expression of HMA genes is tightly regulated by histone modifications. Heavy metal stress induces the enrichment of transcriptional activation mark *H3K4me3* at HMA promoters to upregulate gene expression, while *H3K27me3* acts as a negative regulator to avoid excessive transcription [134]. HMA proteins transport cytoplasmic metal-chelate complexes into vacuoles to reduce cytotoxicity.

**Figure 1:**
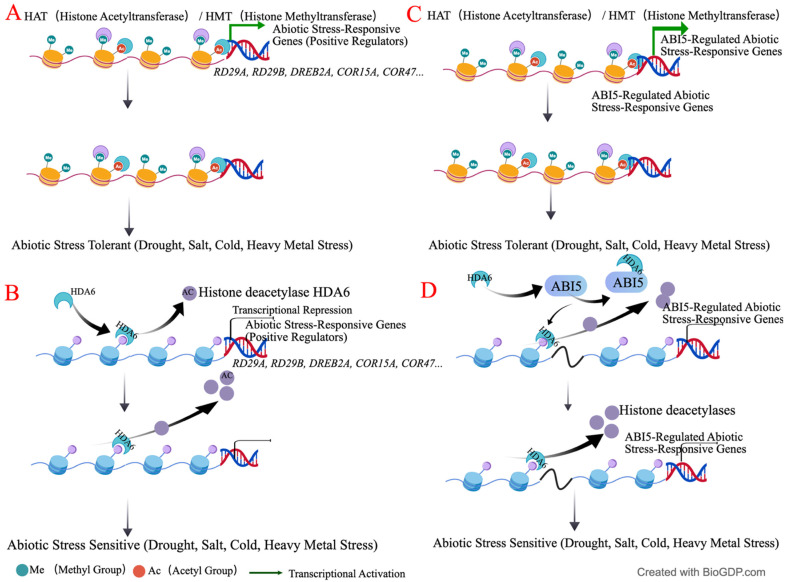
Schematic diagram of the regulatory mechanism of histone modifications in response to abiotic stresses in plants. Note: (**A**,**C**) Positive regulatory pathways: Histone acetyltransferases (HATs) and histone methyltransferases (HMTs) catalyze histone acetylation (Ac) and methylation (Me) modifications at the promoters of abiotic stress-responsive genes (**A**) and ABI5-regulated abiotic stress response genes (**C**), respectively. These modifications loosen chromatin structure, activate gene transcription, and ultimately enhance plant tolerance to multiple abiotic stresses, including drought, salinity, cold, and heavy metals. (**B**,**D**) Negative regulatory pathways: Histone deacetylase *HDA6* directly mediates histone deacetylation of positive abiotic stress regulators (**B**), or interacts with the core ABA signaling transcription factor ABI5 to trigger deacetylation of ABI5-dependent stress-responsive genes (**D**). Deacetylation induces chromatin condensation, represses gene transcription, and reduces abiotic stress tolerance, thereby rendering plants more sensitive to adverse conditions.

In contrast, HDACs function as negative regulators. By removing acetyl groups from histones, HDACs promote chromatin condensation and repress gene transcription [84]. This inhibitory effect maintains cellular energy homeostasis and prevents excessive activation of defense responses under stress, since sustained defense activation consumes substantial energy and impairs normal plant growth and development. Homologs of rice *OsHDA716* also function in heavy metal stress responses, maintaining cellular energy balance by restraining overactive defense pathways [143]. Collectively, histone acetylation and methylation constitute a complete epigenetic cascade linking extracellular adsorption (*PME*/*XTH*), intracellular chelation (*PCS*), and vacuolar compartmentalization (*HMA*) during plant responses to heavy metal stress. The dual regulatory mechanisms mediated by histone acetylation/deacetylation and methylation are summarized in Figure 1.

## 4. Regulatory Mechanisms of Histone Modifications in ABA, CBF/DREB, and Signaling Pathways

Abiotic stress responses in plants are governed by complex signaling networks. As crucial epigenetic regulators, histone modifications extensively interact with multiple core signaling pathways to precisely modulate stress-related gene expression. This section mainly elaborates on the molecular mechanisms underlying the crosstalk between histone modifications and ABA, CBF/DREB, as well as ROS signaling pathways under adverse conditions.

### 4.1. Interactive Mechanisms Between Histone Modifications and the ABA Signaling Pathway

Among the signal transduction pathways involved in plant responses to abiotic stresses, the ABA pathway is one of the most crucial hormonal signaling pathways. ABA accumulates rapidly under stresses such as drought and salinity, activates the *SnRK2* kinase family, and subsequently phosphorylates downstream transcription factors (e.g., the bZIP family), ultimately inducing the expression of numerous stress-responsive genes [144,145]. Histone modifications play dual roles as “signal amplifiers” and “transcriptional gate switches” in this pathway.

Studies have demonstrated that ABA signaling can directly or indirectly regulate the activities of histone acetyltransferases (HATs) and histone deacetylases (HDACs). In rice, *OsHDA716* interacts with the ABA signaling pathway. It deacetylates the pathway transcription factor *OsbZIP46* and works with *OsPUB75* to promote its degradation, forming a negative feedback loop to avoid overactive stress responses. This demonstrates that HDACs can directly regulate the stability of core signaling proteins beyond chromatin modification [9,85].

### 4.2. Coordinated Regulation of Histone Modifications and CBF/DREB Signaling Pathway Under Cold Stress

Histone methylation is also deeply involved in the regulation of signaling cascades. As a potent transcriptional activation mark, *H3K4me3* is abundantly enriched in the promoter regions of actively transcribed genes [13,94]. ABA acts as an upstream regulator of the CBF cascade and dynamically modulates the activities of histone acetyltransferases (HATs) and histone deacetylases (HDACs) [146,147]. ABA signaling enhances the catalytic activity of HATs to elevate histone acetylation levels at CBF loci, while repressing the function of HDACs [25,148]. This synergistic effect relaxes chromatin structure and ultimately triggers the transcriptional activation of the CBF pathway under cold stress [149]. During plant cold stress adaptation, the CBF/DREB transcription factor family functions as a central regulatory node to control cold tolerance capacity [150,151]. Under normal growth conditions, the CBF loci are maintained in a dynamic repressive state dominated by the repressive histone mark *H3K27me3*, which acts synergistically with transcriptional repressors and histone deacetylases to sustain low basal expression levels [13,112,152].

Upon perception of low-temperature signals, JmjC family histone demethylases are rapidly activated and participate in the dynamic removal of the repressive *H3K27me3* mark at the CBF gene promoter. This process is not a direct, instantaneous action of a single enzyme but is part of a multilayered, spatiotemporally specific epigenetic regulatory network, which ultimately relieves transcriptional repression and activates the CBF-mediated cold response pathway [13,110,153]. Meanwhile, histone methyltransferases (HMTs) deposit the activating modification *H3K4me3* at the same chromatin regions [13], accompanied by elevated H3/H4 acetylation levels [154]. The synergistic effect of these multiple activating modifications substantially promotes chromatin relaxation, ensures the rapid and robust transcription of CBF genes, and further triggers the global cold-responsive gene network [94,101,111]. Such histone modification-driven “silencing-to-activation” transition constitutes an essential molecular foundation for the rapid and precise activation of plant stress signaling pathways.

### 4.3. Coordinated Regulation Between Histone Modifications and the ROS Signaling Pathway

Histone modifications also interact closely with the reactive oxygen species (ROS) signaling pathway. Abiotic stress frequently triggers excessive ROS accumulation in plant cells. Beyond acting as toxic by-products of stress injury, ROS function as critical signaling molecules to mediate stress adaptation [155,156]. Accumulating evidence has demonstrated that the R2R3-MYB transcription factor *SiMYB77* from sesame enhances drought and salt tolerance in transgenic tobacco by maintaining stable osmolyte accumulation and ROS homeostasis [122]. The expression of such transcription factors and the activation of their downstream target genes are tightly modulated by the combined effects of *H3K4me3* and histone acetylation. Stress-induced ROS signals can alter the activity of HATs/HDACs and HMTs/HDMs, thereby reshaping the histone modification landscape of stress-related genes. This epigenetic reprogramming further regulates the transcription of antioxidant enzyme systems, including SOD, CAT, and APX, and ultimately maintains intracellular ROS homeostasis under adverse conditions.

Excess ROS accumulated under stress can induce oxidative modification of HATs and HMTs. Such post-translational modifications alter the catalytic efficiency and chromatin-binding capacity of these enzymes, triggering the genome-wide redistribution of histone acetylation and methylation marks. By remodeling the global histone landscape, plants reprogram the transcription of numerous stress-responsive genes and reshape their stress response patterns [157,158,159].

From the perspective of chromatin regulation, HATs/HDACs and HMTs/HDMs, together with core histone marks (*H3K4me3*, *H3K27me3*, and histone acetylation), serve as the key convergence nodes of ABA, CBF, and ROS signaling networks. All three signaling pathways converge on these histone-modifying enzymes and canonical chromatin marks to jointly regulate the transcription of downstream stress genes, forming an integrated epigenetic regulatory network for plant abiotic stress responses [99,160,161].

## 5. Transgenerational Inheritances of Histone Modification Variations and Their Regulatory Mechanisms

### 5.1. Transgenerational Inheritance of Histone Modification Variations Under Abiotic Stress

In natural habitats, plants are frequently exposed to recurring abiotic stresses and have consequently evolved sophisticated stress memory mechanisms to cope with environmental fluctuations [162]. Environmentally induced chromatin marks can be stably transmitted through cell division, enabling the formation of acquired environmental memory, defined as plant epigenetic memory. Such epigenetic regulation empowers plants to rapidly adapt to fluctuating growth conditions [163]. Based on the transmission range, epigenetic memory is categorized into somatic memory and transgenerational memory. Somatic memory functions only within the stressed generation, whereas transgenerational memory can be stably inherited by progeny even after stress cessation [164].

After exposure to drought, salinity, or extreme cold, plants undergo dynamic alterations in histone acetylation and methylation across the genome. Most stress-induced histone modifications mediate somatic stress memory and only function in the stressed generation without heritability. Only a subset of *H3K4me3* and *H3K27me3* can be transmitted via gametes to F1 progeny and establish transgenerational epigenetic memory. Representative marks such as *H3K4me3* and *H3K27me3* can be transmitted via gametes to establish transgenerational epigenetic memory, thereby enabling progeny to respond more rapidly to recurring stress [8]. For instance, in cucumber, cold acclimation sustains elevated *H3K4me3* levels at stress-inducible genes throughout the recovery stage, which is tightly associated with their persistent high transcription. In tobacco, reduced *H3K27me3* deposition at the promoter of *CYP82E4*, a key gene responsible for nicotine conversion, facilitates high gene expression and metabolic transformation [117]. Parental cold-induced *H3K27me3* predominantly establishes cold-specific transgenerational adaptation in plants, instead of conferring universal broad-spectrum resistance to diverse abiotic stresses [165,166,167]. As a canonical repressive mark catalyzed by the *PRC2* complex, *H3K27me3* silences developmental and stress-related genes under normal growth conditions [4,168]. Under cold stress, JUMONJI family demethylases specifically eliminate *H3K27me3* at the loci of CBF and COR genes to release transcriptional repression and form cold memory [169,170]. This epigenetic reprogramming is highly locus-specific and barely affects the regulatory regions of ABA signaling, osmotic adjustment, and salt genes [106,123]. Plants can develop cross-tolerance mediated by shared signaling molecules, including ROS and ABA, but such phenotypic changes are not driven by *H3K27me3* modification [171]. Genome-wide studies on cucumber and grape further confirm that cold-induced *H3K27me3* redistribution is restricted to cold-adaptive genes, without large-scale de-repression of drought or salt-responsive genes [172,173]. In addition, partial epigenetic reset during plant sexual reproduction limits the stable inheritance of *H3K27me3*-mediated broad stress resistance [132,174]. Notably, asymmetric stress priming has been reported: parental drought treatment improves cold tolerance of wheat progeny, while the reverse effect has not been observed, which further verifies the specificity of *H3K27me3*-related stress memory [175]. To address this open scientific question, future research needs to combine phenotypic identification, ChIP-seq, RNA-seq, and genetic analysis using *PRC2* or *H3K27me3* demethylase mutants [176]. At present, the academic community generally agrees that cold-induced *H3K27me3* changes mainly serve cold adaptation, and cannot be regarded as a general mechanism for plant broad-spectrum abiotic stress resistance [167,177]. These findings indicate that stress can activate plant defense pathways by erasing the *H3K27me3* repressive brake from stress-related loci.

Stress-induced reprogramming of histone modifications constitutes the molecular basis of transgenerational epigenetic memory. Accumulating studies have demonstrated that such memory phenomena are widespread in multiple plant species, including Arabidopsis, rice, and cotton. Analysis of cotton seedlings subjected to recurrent drought stress has revealed distinct “memory signatures” in physiological indexes, transcriptomic profiles, and histone modification patterns. Plants primed by previous stress exhibited faster and stronger responses upon secondary drought exposure [178]. Similarly, relevant studies in rice have confirmed that drought stress triggers long-lasting biochemical and epigenetic alterations to establish stable stress memory [179]. The formation of epigenetic stress memory represents a conserved adaptive strategy shaped during plant evolution, which enables progeny to gain a survival advantage under foreseeable adverse environments [180]. Collectively, these findings provide solid evidence for the transgenerational inheritance of histone modifications and highlight the considerable research value and application potential of epigenetic variation in crop stress resistance improvement. Furthermore, obvious differences exist in transgenerational memory among distinct stress types and plant species. Cold-induced epigenetic memory generally has stronger stability and longer maintenance time compared with drought and salt stress. In addition, model plants such as Arabidopsis show more obvious heritable effects, while plateau crops like hulless barley and sea buckthorn exhibit relatively weaker transgenerational stress memory and unique transmission rules. Current studies on transgenerational epigenetic memory are mainly based on model plants. Whether the stress memory patterns of plateau crops are consistent with model species, and how to artificially regulate epigenetic memory to improve crop durability of stress resistance, are key unresolved directions for future research.

### 5.2. Regulatory Mechanisms Underlying Transgenerational Inheritance of Histone Modifications

Unlike mammals, which undergo extensive epigenetic reprogramming during germ cell formation and early embryonic development, plants lack such a thorough genome-wide resetting. Consequently, somatic epigenetic modifications acquired during the parental life cycle, including histone marks, are more readily transmitted to progeny through gametes. In Arabidopsis, accumulating evidence demonstrates that histone H3 lysine 27 trimethylation (*H3K27me3*) is not completely erased during gametogenesis and fertilization. This repressive modification is stably retained at centromeric regions, transposon-enriched loci, and the promoters of certain developmentally regulated genes [181]. The transgenerational inheritance of plant histone modifications relies not on the independent transmission of a single chromatin mark, but on a sophisticated regulatory network integrated by multiple epigenetic pathways, core enzyme complexes, and environmental signals. The RNA-directed DNA methylation (*RdDM*) pathway, POLYCOMB repressive complex 2 (*PRC2*), and abiotic stress induction function as central modules within this regulatory system. These three components act synergistically and hierarchically to ensure the stable maintenance and generational transmission of histone modifications, thereby establishing the essential molecular foundation for the formation of plant stress memory. RNA-directed DNA methylation (*RdDM*)-mediated regulation: The *RdDM* pathway is indispensable for the epigenetic silencing of transposable elements and repetitive sequences in plants [182]. Guided by small RNAs, particularly 24-nt siRNAs, this pathway establishes DNA methylation and acts synergistically with histone methylation marks such as H3K9me2 and *H3K27me3* to stabilize transcriptional silencing, which can be stably inherited across generations [183,184]. By recruiting the de novo methyltransferase DRM2, the *RdDM* pathway catalyzes cytosine methylation in all sequence contexts (CG, CHG, and CHH) and is especially critical for the establishment and maintenance of asymmetric CHH methylation. Polycomb repressive complex 2 (*PRC2*) serves as the core enzyme complex responsible for *H3K27me3* deposition [185,186]. *PRC2* recognizes and binds to existing *H3K27me3*, and further propagates this repressive mark to adjacent nucleosomes, forming a positive feedback loop that ensures the stable retention and spreading of *H3K27me3* [186]. In Arabidopsis, *PRC2* plays a vital role in plant regeneration, and its functional dynamics directly influence the transgenerational transmission of histone modifications [185]. To be specific, many histone marks will be erased during plant gametogenesis and fertilization for normal developmental reprogramming. However, stress-related *H3K27me3* and *H3K4me3* can be protected by *PRC2* and *RdDM* complexes, which shield these epigenetic marks from large-scale epigenetic reset, thus realizing stable transgenerational transmission.

As sessile species, plants are unable to flee harsh environments and have developed elaborate regulatory mechanisms to sense and cope with various abiotic stressors [187]. Environmental stimuli, including cold, heat, drought, and salinity, trigger extensive epigenetic alterations, especially dynamic changes in histone modifications. These stress-induced epigenetic alterations can be partially transmitted to subsequent generations, enabling progeny to mount faster and stronger responses to recurring environmental challenges and thereby forming stable “stress memory”. For example, prolonged cold stress induces heritable genomic and epigenomic variations, such as altered DNA methylation patterns, in Arabidopsis offspring [174]. High-temperature stress also triggers global changes in *H3K4me3* and *H3K27me3* levels in cotton, ultimately affecting pollen fertility [188,189].

## 6. Applications of Histone Modification Regulation in Bioengineering

### 6.1. CRISPR/Cas9-Based Editing of Histone Modification Enzyme Genes

Regulation of histone modification enzymes based on gene editing represents an efficient technical strategy for improving crop stress resistance. The CRISPR/Cas9 system provides unprecedented precision for such epigenetic manipulation and lays a solid technical foundation for the breeding of stress-tolerant crops [98,190]. Guided by single-guide RNA (sgRNA), the Cas9 nuclease cleaves target genomic sites with high accuracy, and subsequent endogenous DNA repair mechanisms enable targeted gene knockout, insertion, or replacement [191]. Due to its high efficiency and unique specificity, CRISPR/Cas9 has been widely applied to enhance crop tolerance against drought, salinity, extreme temperature, and other abiotic stresses [192]. Compared with time-consuming and low-efficiency conventional breeding, this technology facilitates the rapid creation of stress-resistant germplasm and helps mitigate the severe threat of abiotic stress to global food security. Serving as a crucial epigenetic regulatory mode, histone modifications modulate gene expression without altering the underlying DNA sequence, thereby orchestrating plant growth, development, and stress adaptation. Histone acetylation and deacetylation, two dynamic and reversible processes catalyzed by histone acetyltransferases (HATs) and histone deacetylases (HDACs), jointly determine chromatin accessibility and govern transcriptional activity. This regulatory characteristic provides solid theoretical and physiological support for editing histone modification enzyme genes via CRISPR/Cas9, offering a promising route to epigenetically improve crop abiotic stress resistance.

### 6.2. Applications of dCas9-Based Epigenetic Editing Tools in the Regulation of Histone Modifications

In plants, the activation of drought-resistance genes, including *DREB2A*, *AREB1*, and *RD29A*, is essential for enhancing plant survival under water-deficit conditions [193]. Conventional genetic manipulation approaches frequently cause off-target effects and developmental defects. In contrast, the dCas9-HAT system can precisely target the promoter or enhancer regions of these stress-responsive genes, induce local histone acetylation, and thereby specifically activate their transcription [194]. Such targeted epigenetic modification effectively strengthens drought response capacity and improves overall abiotic stress tolerance.

For example, Roca Paixão et al. constructed a dCas9-HAT fusion that specifically targeted the promoter of the key drought-resistance gene *AREB1* in *Arabidopsis*. The system significantly increased H3K27 acetylation at the *AREB1* promoter, upregulated *AREB1* and its downstream gene *RD29A*, and consequently improved drought tolerance: transgenic plants exhibited higher chlorophyll content, faster stomatal closure, and higher survival rates under water deficit [195]. Similarly, Lee et al. developed a modular MS2-dCas9 toolkit capable of recruiting different epigenetic effectors, including the H3K27 acetyltransferase p300 and the H3K9 methyltransferase KYP, to target loci. When delivered to the floral regulator FT, the system induced specific histone modifications and altered flowering time, demonstrating its utility for targeted epigenetic reprogramming in plants [196]. This editing strategy circumvents the pleiotropic abnormalities and off-target risks commonly induced by constitutive overexpression. Meanwhile, it enables flexible spatial and temporal control of gene expression, showing prominent advantages for crop stress improvement.

## 7. Conclusions and Perspective

In summary, histone modifications serve as the core epigenetic regulatory hub for plants to perceive, transmit, and adapt to abiotic stresses. Existing research on plateau crops has obvious shortcomings. First, the absence of tissue-specific epigenetic profiles is mainly attributed to the limitations of conventional epigenomic methods, which can be solved preferentially. Second, the crosstalk of multiple coexisting stresses involves intertwined regulatory networks, resulting in moderate research difficulty. Third, the large-scale field application of epigenetic editing is hindered by imperfect supporting technologies and harsh plateau field conditions, making it the most challenging long-term research task. Nevertheless, systematic analysis and field validation regarding the stability, transmission efficiency, and detailed molecular mechanisms underlying the transgenerational inheritance of histone modifications are still insufficient.

The Qinghai–Tibet Plateau features a unique environment characterized by the superposition of multiple abiotic stresses, including extreme low temperatures, drought, salinization, and heavy metals in mining areas. Conducting research on histone modification-mediated stress adaptation mechanisms in plateau plants in this region not only possesses distinct regional characteristics but also holds significant theoretical innovation value. With the in-depth study of epigenetic memory and chromatin regulatory mechanisms, epigenetic precision breeding based on histone modifications has become a new direction for improving crop stress resistance. Combining CRISPR/Cas9 gene editing with dCas9-targeted epigenetic editing technology enables site-specific and spatiotemporally specific regulation of histone modifications, effectively enhancing plant stress resistance without altering the genome sequence, accelerating the breeding of new varieties adapted to extreme environments, and greatly shortening the traditional breeding cycle.

Future research should prioritize three targeted and innovative research directions tailored to plateau agricultural environments. First, uncover the molecular crosstalk among different histone modifications and their interplay with core stress signaling pathways under the combined cold, drought, salinity, and heavy metal stresses of the Qinghai–Tibet Plateau. Second, integrate single-cell epigenomic approaches to build high-resolution tissue- and cell-specific histone modification maps for representative plateau crops, and explore the molecular basis of their unique stress adaptation strategies. Third, optimize CRISPR/dCas9-mediated epigenetic editing systems adapted to plateau field conditions, and conduct long-term field verification and comprehensive biosafety evaluation to facilitate the practical application of precision epigenetic breeding. It should be noted that current CRISPR/dCas9-based epigenetic editing still has prominent technical constraints: off-target mutations, poor tissue-specific delivery performance, and the difficulty in maintaining stable inheritance of edited traits across generations. For future field popularization, systematic risk assessment and long-term monitoring are required to ensure ecological and agricultural safety.

Critically, most current investigations focus on phenotypic observations and single-stress response mechanisms, while mechanistic explanations for compound stresses in plateau environments remain insufficient. A number of conflicting conclusions have been reported regarding the substrate preference of modification enzymes and the inheritance of epigenetic stress memory. Clarifying these academic divergences, exploring the inherent laws behind disputed results, and addressing the above key scientific challenges will be the core focus of subsequent research in this field.

## Data Availability

No new data were created or analyzed in this study.

## References

[1] Shakespear S., Sivaji M., Kumar V., Arumugam Pillai M., Wani S.H., Penna S., Yasin J.K. (2024). Navigating through harsh conditions: Coordinated networks of plant adaptation to abiotic stress. J. Plant Growth Regul..

[2] Akhter Z., Bi Z.Z., Ali K., Sun C., Fiaz S., Haider F.U., Bai J.P. (2021). In response to abiotic stress, DNA methylation confers epigenetic changes in plants. Plants.

[3] Zhang P.X., Huang S.W. (2025). Transgenerational epigenetic inheritance of cold adaptation in rice: Evidence for neo-Lamarckian concepts. Mol. Plant.

[4] Ma L.J., Xing L.H., Li Z.C., Jiang D.H. (2025). Epigenetic control of plant abiotic stress responses. J. Genet. Genom..

[5] Kornberg R.D. (1974). Chromatin structure: A repeating unit of histones and DNA. Science.

[6] Gujral P., Mahajan V., Lissaman A.C., Ponnampalam A.P. (2020). Histone acetylation and the role of histone deacetylases in normal cyclic endometrium. Reprod. Biol. Endocrinol..

[7] Li X.Y., Yu J.T., Dong Y.H., Shen X.Y., Hou R., Xie M.M., Wei J., Hu X.W., Dong Z.H., Shan R.R. (2023). Protein acetylation and related potential therapeutic strategies in kidney disease. Pharmacol. Res..

[8] Wang F., Li C.H., Liu Y., He L.F., Li P., Guo J.X., Zhang N., Zhao B., Guo Y.D. (2024). Plant responses to abiotic stress regulated by histone acetylation. Front. Plant Sci..

[9] Sun Y., Gu X.Y., Qu C.F., Jin N., Qin T., Jin L., Huang J.L. (2025). *OsPUB75–OsHDA716* mediates deactivation and degradation of *OsbZIP46* to negatively regulate drought tolerance in rice. Plant Physiol..

[10] Seni S., Singh R.K., Prasad M. (2023). Dynamics of epigenetic control in plants via SET domain containing proteins: Structural and functional insights. Biochim. Biophys. Acta Gene Regul. Mech..

[11] Xiao J., Lee U.S., Wagner D. (2016). Tug of war: Adding and removing histone lysine methylation in *Arabidopsis*. Curr. Opin. Plant Biol..

[12] He K.X., Cao X.F., Deng X. (2021). Histone methylation in epigenetic regulation and temperature responses. Curr. Opin. Plant Biol..

[13] Faivre L., Kinscher N.F., Kuhlmann A.B., Xu X., Kaufmann K., Schubert D. (2024). Cold stress induces rapid gene-specific changes in the levels of *H3K4me3* and *H3K27me3* in *Arabidopsis thaliana*. Front. Plant Sci..

[14] An B., Cai H., Li B., Zhang S., He Y., Wang R., Jiao C., Guo Y., Xu L., Xu Y. (2023). Molecular evolution of histone methylation modification families in the plant kingdom and their genome-wide analysis in barley. Int. J. Mol. Sci..

[15] Soupsana K., Karanika E., Kiosse F., Christogianni A., Sfikas Y., Topalis P., Batistatou A., Kanaki Z., Klinakis A., Politou A.S. (2021). Distinct roles of haspin in stem cell division and male gametogenesis. Sci. Rep..

[16] Zhou H.P., Shi H.F., Yang Y.Q., Feng X.X., Chen X., Xiao F., Lin H.H., Guo Y. (2024). Insights into plant salt stress signaling and tolerance. J. Genet. Genom..

[17] Chen Z.X., Ye C.J., Zeng Y., Guo J., Zhou X.Q., Chen D.G., Liu J., Liu C.G., Jaremko M., Chen K. (2025). Multiomics analysis revealed the temporally common and specific molecular changes in *Arabidopsis thaliana* (L.) under salt stress. BMC Genom..

[18] Yung W.S., Li M.W., Sze C.C., Wang Q.W., Lam H.-M. (2021). Histone modifications and chromatin remodelling in plants in response to salt stress. Physiol. Plant..

[19] Singha R., Mahajan M., Das S., Kumar V. (2023). Protein SUMOylation: Current updates and insights to elucidate potential roles of SUMO in plants. S. Afr. J. Bot..

[20] Boulanger M., Chakraborty M., Tempé D., Piechaczyk M., Bossis G. (2021). SUMO and transcriptional regulation: The lessons of large-scale proteomic, modifomic and genomic studies. Molecules.

[21] Gao S., Zeng X., Wang J., Xu Y., Yu C., Huang Y., Wang F., Wu K., Yang S. (2021). *Arabidopsis* SUMO E3 Ligase *SIZ1* Interacts with *HDA6* and Negatively Regulates *HDA6* Function during Flowering. Cells.

[22] Han D.L., Chen C., Xia S.M., Liu J., Shu J., Nguyen V., Lai J.B., Cui Y.H., Yang C.W. (2021). Chromatin-associated SUMOylation controls the transcriptional switch between plant development and heat stress responses. Plant Commun..

[23] Huang J., Huang J., Feng Q., Shi Y., Wang F., Zheng K., Huang Q., Jiang J., Luo S., Xie Y. (2023). SUMOylation facilitates the assembly of a nuclear factor-Y complex to enhance thermotolerance in *Arabidopsis*. J. Integr. Plant Biol..

[24] Wang F.G., Liu Y.Y., Shi Y.Q., Han D., Wu Y., Ye W., Yang H., Li G., Cui F., Wan S. (2020). SUMOylation stabilizes the transcription factor *DREB2A* to improve plant thermotolerance. Plant Physiol..

[25] Bajpai S.K., Nisha, Pandita S., Bahadur A., Verma P.C. (2024). Recent advancements in the role of histone acetylation dynamics to improve stress responses in plants. Mol. Biol. Rep..

[26] Singh M., Singh A., Yadav N., Yafav D.K. (2022). Current perspectives of ubiquitination and SUMOylation in abiotic stress tolerance in plants. Front. Plant Sci..

[27] Wang X., Zhang X.Y., Song C.P., Gong Z.Z., Yang S.H., Ding Y.L. (2023). PUB25 and PUB26 dynamically modulate *ICE1* stability via differential ubiquitination during cold stress in Arabidopsis. Plant Cell.

[28] Nunez-Vazquez R., Madeira S., Rodríguez-Casillas L., Gomez-Martinez D., Desvoyes B., Gutierrez C. (2025). The histone variant *H3.14* is an early player in the abiotic stress response of *Arabidopsis*. Dev. Cell.

[29] Miao R.Q., Zhang Y., Liu X.X., Yuan Y., Zang W., Li Z.Q., Yan X.F., Pang Q.Y., Zhang A.Q. (2024). Histone variant *H2A.Z* is required for plant salt response by regulating gene transcription. Plant Cell Environ..

[30] Shi Z., Zhou M.Y., Song W., Liu Y., Wang R.H., Wang Y.D., Zhang R.Y., Zhao J.R., Ren W. (2023). Trash to treasure: Lactate and protein lactylation in maize root impacts response to drought. Sci. China Life Sci..

[31] Contreras-de la Rosa P.A., Aragón-Rodríguez C., Ceja-López J.A., García-Arteaga K.F., De-la-Peña C. (2022). Lysine crotonylation: A challenging new player in the epigenetic regulation of plants. J. Proteom..

[32] Alam N.B., Jain M., Mustafiz A. (2024). Pyramiding D-lactate dehydrogenase with the glyoxalase pathway enhances abiotic stress tolerance in plants. Plant Physiol. Biochem..

[33] Beamer Z.G., Routray P., Choi W.G., Spangler M.K., Lokdarshi A., Roberts D.M. (2021). Aquaporin family lactic acid channel NIP2;1 promotes plant survival under low oxygen stress in *Arabidopsis*. Plant Physiol..

[34] Shi C., Guo F., Sun Y., Han J., Zheng X., Zhang J., Qin C., Tan Z., Lin J., Wang J. (2024). Physiological and transcriptomic analysis of *Hordeum jubatum* seedlings in response to salt, alkali and drought stresses under uniform water potential. Environ. Exp. Bot..

[35] Zhao H., Ni S., Cai S., Zhang G. (2021). Comprehensive dissection of primary metabolites in response to diverse abiotic stress in barley at seedling stage. Plant Physiol. Biochem..

[36] Guo R., Zhou Z., Cai R., Liu L., Wang R., Sun Y., Wang D., Yan Z., Guo C. (2024). Metabolomic and physiological analysis of alfalfa (*Medicago sativa* L.) in response to saline and alkaline stress. Plant Physiol. Biochem..

[37] Kuzmina N.V., Ostapiv R.D., Ostapiv D.D., Golovach P.I. (2024). Features of the lactate dehydrogenase isoenzymes spectrum in animal tissues and organs. Regul. Mech. Biosys..

[38] Meng F., He J., Zhang X., Lyu W., Wei R., Wang S., Du Z., Wang H., Bi J., Hua X. (2025). Histone lactylation antagonizes senescence and skeletal muscle aging by modulating aging-related pathways. Adv. Sci..

[39] Xie Y., Hu H., Liu M., Zhou T., Cheng X., Huang W., Cao L. (2022). The role and mechanism of histone lactylation in health and diseases. Front. Genet..

[40] Minami E., Sasa K., Yamada A., Kawai R., Yoshida H., Nakano H., Maki K., Kamijo R. (2023). Lactate-induced histone lactylation by p300 promotes osteoblast differentiation. PLoS ONE.

[41] Sheng X., Lin H., Cole P.A., Zhao Y. (2025). Biochemistry and regulation of histone lysine l-lactylation. Nat. Rev. Mol. Cell Biol..

[42] Zhang D., Gao J., Zhu Z., Mao Q., Xu Z., Singh P.K., Rimayi C.C., Moreno-Yruela C., Xu S., Li G. (2024). Lysine l-lactylation is the dominant lactylation isomer induced by glycolysis. Nat. Chem. Biol..

[43] Liberti M.V., Locasale J.W. (2020). Histone lactylation: A new role for glucose metabolism. Trends Biochem. Sci..

[44] Wang L., Wang Y., Meng M., Ma N., Wei G., Huo R., Chang G., Shen X. (2023). High-concentrate diet elevates histone lactylation mediated by *p300/CBP* through the upregulation of lactic acid and induces an inflammatory response in mammary gland of dairy cows. Microb. Pathog..

[45] Hu Y., He Z., Li Z., Wang Y., Wu N., Sun H., Zhou Z., Hu Q., Cong X. (2024). Lactylation: The novel histone modification influence on gene expression, protein function, and disease. Clin. Epigenet..

[46] Moreno-Yruela C., Zhang D., Wei W., Bæk M., Liu W., Gao J., Danková D., Nielsen A.L., Bolding J.E., Yang L. (2022). Class I histone deacetylases (HDAC1–3) are histone lysine delactylases. Sci. Adv..

[47] Gonzatti M.B., Hintzen J.C.J., Sharma I., Najar M.A., Tsusaka T., Marcinkiewicz M.M., Da Silva Crispim C.V., Snyder N.W., Burslem G.M., Goldberg E.L. (2025). Class I histone deacetylases catalyze lysine lactylation. J. Biol. Chem..

[48] Tsukihara S., Akiyama Y., Shimada S., Hatano M., Igarashi Y., Taniai T., Tanji Y., Kodera K., Yasukawa K., Umeura K. (2024). Delactylase effects of SIRT1 on a positive feedback loop involving the H19-glycolysis-histone lactylation in gastric cancer. Oncogene.

[49] Zu H., Li C., Dai C., Pan Y., Ding C., Sun H., Zhang X., Yao X., Zang J., Mo X. (2022). SIRT2 functions as a histone delactylase and inhibits the proliferation and migration of neuroblastoma cells. Cell Discov..

[50] Liu R., Wu J., Guo H., Yao W., Li S., Lu Y., Jia Y., Liang X., Tang J., Zhang H. (2023). Post-translational modifications of histones: Mechanisms, biological functions, and therapeutic targets. MedComm.

[51] Li X., Cai P., Tang X., Wu Y., Zhang Y., Rong X. (2024). Lactylation modification in cardiometabolic disorders: Function and mechanism. Metabolites.

[52] Wu H., Huang H., Zhao Y. (2023). Interplay between metabolic reprogramming and post-translational modifications: From glycolysis to lactylation. Front. Immunol..

[53] Mann E.R., Lam Y.K., Uhlig H.H. (2024). Short-chain fatty acids: Linking diet, the microbiome and immunity. Nat. Rev. Immunol..

[54] Zeaiter N., Belot L., Cunin V., Nahed R.A., Tokarska-Schlattner M., Le Gouellec A., Petosa C., Khochbin S., Schlattner U. (2024). Acetyl-CoA synthetase (ACSS2) does not generate butyryl- and crotonyl-CoA. Mol. Metab..

[55] Xie J., Ju J., Zhou P., Chen H., Wang S., Wang K., Wang T., Chen X., Chen Y., Wang K. (2024). The mechanisms, regulations, and functions of histone lysine crotonylation. Cell Death Discov..

[56] Wang M., Lin H. (2021). Understanding the function of mammalian sirtuins and protein lysine acylation. Annu. Rev. Biochem..

[57] Ren X., Zhou Y., Xue Z., Hao N., Li Y., Guo X., Wang D., Shi X., Li H. (2020). Histone benzoylation serves as an epigenetic mark for DPF and YEATS family proteins. Nucleic Acids Res..

[58] Xu M., Luo J., Li Y., Shen L., Zhang X., Yu J., Guo Z., Wu J., Chi Y., Yang J. (2021). First comprehensive proteomics analysis of lysine crotonylation in leaves of peanut (*Arachis hypogaea* L.). Proteomics.

[59] Li K., Wang Z. (2021). Histone crotonylation-centric gene regulation. Epigenetics Chromatin.

[60] Tan M., Luo H., Lee S., Jin F., Yang J.S., Montellier E., Buchou T., Cheng Z.Y., Rousseaux S., Rajagopal N. (2011). Identification of 67 histone marks and histone lysine crotonylation as a new type of histone modification. Cell.

[61] Sabari B.R., Tang Z.Y., Huang H., Yong-Gonzalez V., Molina H., Kong H.E., Dai L.Z., Shimada M., Cross J.R., Zhao Y.M. (2015). Intracellular crotonyl-coa stimulates transcription through p300-catalyzed histone crotonylation. Mol. Cell.

[62] Nunez-Vazquez R., Desvoyes B., Gutierrez C. (2022). Histone variants and modifications during abiotic stress response. Front. Plant Sci..

[63] Vivek Hari Sundar G., Madhu A., Archana A., Shivaprasad P.V. (2024). Plant histone variants at the nexus of chromatin readouts, stress and development. Biochim. Biophys. Acta-Gen. Subj..

[64] Chen Z., Ponts N. (2020). *H2A.Z* and chromatin remodelling complexes: A focus on fungi. Crit. Rev. Microbiol..

[65] Sureshkumar S., Balasubramanian S. (2021). Complexes and complexities: *INO80* takes center stage. Mol. Plant.

[66] Martire S., Banaszynski L.A. (2020). The roles of histone variants in fine-tuning chromatin organization and function. Nat. Rev. Mol. Cell Biol..

[67] Do B.H., Nguyen N.H. (2024). *H2A.Z* removal mediates the activation of genes accounting for cell elongation under light and temperature stress. Plant Cell Rep..

[68] Yang Y., Zhang L., Xiong C., Chen J., Wang L., Wen Z., Yu J., Chen P., Xu Y., Jin J. (2021). HIRA complex presets transcriptional potential through coordinating depositions of the histone variants H3.3 and *H2A.Z* on the poised genes in mESCs. Nucleic Acids Res..

[69] Tiwari A., Pandey-Rai S., Rai K.K., Tiwari A., Pandey N. (2022). Molecular and epigenetic basis of heat stress responses and acclimatization in plants. Nucleus.

[70] Gandhivel V.H.S., Sotelo-Parrilla P., Raju S., Jha S., Gireesh A., Harshith C.Y., Gut F., Vinothkumar K.R., Berger F., Jeyaprakash A.A. (2025). An Oryza-specific histone H4 variant predisposes H4 lysine 5 acetylation to modulate salt stress responses. Nat. Plants.

[71] Li X., Mei Q., Yu Q., Wang M., He F., Xiao D., Liu H., Ge F., Yu X., Li S. (2023). The *TORC1* activates Rpd3L complex to deacetylate *Ino80* and *H2A.Z* and repress autophagy. Sci. Adv..

[72] Guarino F., Cicatelli A., Castiglione S., Agius D.R., Orhun G.E., Fragkostefanakis S., Leclercq J., Dobránszki J., Kaiserli E., Lieberman-Lazarovich M. (2022). An epigenetic alphabet of crop adaptation to climate change. Front. Genet..

[73] Agudelo Garcia P.A., Nagarajan P., Parthun M.R. (2020). Hat1-dependent lysine acetylation targets diverse cellular functions. J. Proteome Res..

[74] Matsushita N. (2023). Dysregulated histone acetylation causes congenital diseases. Gene Rep..

[75] Fina J.P., Masotti F., Rius S.P., Crevacuore F., Casati P. (2017). HAC1 and HAF1 histone acetyltransferases have different roles in uv-b responses in *Arabidopsis*. Front. Plant Sci..

[76] Earley K.W., Shook M.S., Brower-Toland B., Hicks L., Pikaard C.S. (2007). *In vitro* specificities of Arabidopsis co-activator histone acetyltransferases: Implications for histone hyperacetylation in gene activation. Plant J..

[77] Li H., Liu H., Pei X., Chen H.Y., Li X., Wang J.R., Wang C.Y. (2021). Comparative genome-wide analysis and expression profiling of histone acetyltransferases and histone deacetylases involved in the response to drought in wheat. J. Plant Growth Regul..

[78] Gan L., Wei Z., Yang Z., Li F.G., Wang Z. (2021). Updated mechanisms of GCN5—The monkey king of the plant kingdom in plant development and resistance to abiotic stresses. Cells.

[79] Li S., He X., Gao Y., Zhou C.G., Chiang V.L., Li W. (2021). Histone acetylation changes in plant response to drought stress. Genes.

[80] Chhatwal H., Naik J., Pandey A., Trivedi P.K. (2024). Broadening the epigenetic horizon of abiotic stress response in plants. Plant Growth Regul..

[81] Feng P., Sun X., Liu X., Li Y., Sun Q., Lu H., Li M., Ding X., Dong Y. (2022). Epigenetic regulation of plant tolerance to salt stress by histone acetyltransferase GsMYST1 from wild soybean. Front. Plant Sci..

[82] Ivanova T., Dincheva I., Badjakov I., Iantcheva A. (2023). Transcriptional and metabolic profiling of *Arabidopsis thaliana* transgenic plants expressing histone acetyltransferase *HAC1* upon the application of abiotic stress—Salt and low temperature. Metabolites.

[83] McCarthy R.L., Kaeding K.E., Keller S.H., Zhong Y., Xu L.Q., Hsieh A., Hou Y., Donahue G., Becker J.S., Alberto O. (2021). Diverse heterochromatin-associated proteins repress distinct classes of genes and repetitive elements. Nat. Cell Biol..

[84] Du Q., Fang Y., Jiang J., Chen M., Fu X., Yang Z., Luo L., Wu Q., Yang Q., Wang L. (2022). Characterization of histone deacetylases and their roles in response to abiotic and PAMPs stresses in Sorghum bicolor. BMC Genom..

[85] Sun Y., Xie Z.Z., Jin L., Qin T., Zhan C.H., Huang J.L. (2024). Histone deacetylase OsHDA716 represses rice chilling tolerance by deacetylating *OsbZIP46* to reduce its transactivation function and protein stability. Plant Cell.

[86] Baek D., Shin G., Kim M.C., Shen M.Z., Lee S.Y., Yun D.J. (2020). Histone deacetylase HDA9 with ABI4 contributes to abscisic acid homeostasis in drought stress response. Front. Plant Sci..

[87] Zheng Y., Ge J., Bao C., Chang W., Liu J., Shao J., Liu X., Su L., Pan L., Zhou D.X. (2020). Histone deacetylase HDA9 and WRKY53 transcription factor are mutual antagonists in regulation of plant stress response. Mol. Plant.

[88] Aquea F., Timmermann T., Arce-Johnson P. (2010). Analysis of histone acetyltransferase and deacetylase families of *Vitis vinifera*. Plant Physiol. Biochem..

[89] Luo M., Wang Y.Y., Liu X., Yang S., Wu K. (2012). HD2 proteins interact with RPD3-type histone deacetylases. Plant Signal. Behav..

[90] Cui X.Y., Dard A., Reichheld J.P., Zhou D.X. (2023). Multifaceted functions of histone deacetylases in stress response. Trends Plant Sci..

[91] Yu M.H., Liao W.C., Wu K. (2025). Histone methylation in plant responses to abiotic stresses. J. Exp. Bot..

[92] Xia H., Zhang Y.T., Chen X., Zeng X.L., Cai X., Li Z.Q., Chen H.G., Yang J., Zou J.J. (2024). Genome-wide identification of Osmanthus fragrans histone modification genes and analysis of their expression during the flowering process and under azacytidine and ethylene treatments. Plants.

[93] Julian R., Patrick R.M., Li Y. (2023). Organ-specific characteristics govern the relationship between histone code dynamics and transcriptional reprogramming during nitrogen response in tomato. Commun. Biol..

[94] Zeng Z., Zhang W., Marand A.P., Zhu B., Buell C.R., Jiang J. (2019). Cold stress induces enhanced chromatin accessibility and bivalent histone modifications *H3K4me3* and *H3K27me3* of active genes in potato. Genome Biol..

[95] Sena S., Prakash A., Johannes V.S., Kumar V. (2024). Epigenetic control of plant regeneration: Unraveling the role of histone methylation. Curr. Plant Biol..

[96] Li J., Li Q., Wang W., Zhang X., Chu C., Tang X., Zhu B., Xiong L., Zhao Y., Zhou D.X. (2023). DELLA-mediated gene repression is maintained by chromatin modification in rice. EMBO J..

[97] Zhao W., Wang X., Zhang Q., Zheng Q., Yao H., Gu X., Liu D., Tian X., Wang X., Li Y. (2022). H3K36 demethylase *JMJ710* negatively regulates drought tolerance by suppressing MYB48-1 expression in rice. Plant Physiol..

[98] Vincent S.A., Kim J.M., Perez-Salamo I., To T.K., Torii C., Ishida J., Tanaka M., Endo T.A., Bhat P., Devlin P.F. (2022). Jasmonates and histone deacetylase 6 activate arabidopsis genome-wide histone acetylation and methylation during the early acute stress response. BMC Biol..

[99] Chang Y.N., Zhu C., Jiang J., Zhang H., Zhu J.K., Duan C.G. (2020). Epigenetic regulation in plant abiotic stress responses. J. Integr. Plant Biol..

[100] Hirakawa T., Tanno S., Ohara K. (2023). N-acetylglutamic acid alleviates oxidative stress based on histone acetylation in plants. Front. Plant Sci..

[101] Wang J., Liang Y., Gong Z., Zheng J., Li Z., Zhou G., Xu Y., Li X. (2024). Genomic and epigenomic insights into the mechanism of cold response in upland cotton (*Gossypium hirsutum*). Plant Physiol. Biochem..

[102] Hou Y., Lu Q., Su J., Jin X., Jia C., An L., Tian Y., Song Y. (2022). Genome-wide analysis of the HDAC gene family and its functional characterization at low temperatures in tartary buckwheat (*Fagopyrum tataricum*). Int. J. Mol. Sci..

[103] Abdulraheem M.I., Xiong Y., Moshood A.Y., Cadenas-Pliego G., Zhang H., Hu J. (2024). Mechanisms of plant epigenetic regulation in response to plant stress: Recent discoveries and implications. Plants.

[104] Bai J.T., Shi Z.Y., Zheng S.Z. (2023). The role of histone modifications in heat signal transduction in plants. Adv. Biol..

[105] Park M., Williams D.S., Turpin Z.M., Wiggins Z.J., Tsolova V.M., Onokpise O.U., Bass H.W. (2021). Differential nuclease sensitivity profiling uncovers a drought responsive change in maize leaf chromatin structure for two large retrotransposon derivatives, Uloh and Vegu. Plant Direct.

[106] Adel S., Carels N. (2023). Plant tolerance to drought stress with emphasis on wheat. Plants.

[107] Siddique A.B., Parveen S., Rahman M.Z., Rahman J. (2024). Revisiting plant stress memory: Mechanisms and contribution to stress adaptation. Physiol. Mol. Biol. Plants.

[108] Shilpa, Thakur R., Prasad P. (2024). Epigenetic regulation of abiotic stress responses in plants. Biochim. Biophys. Acta Gen. Subj..

[109] Zhang H., Lang Z.B., Zhu J.K., Wang P.C. (2025). Tackling abiotic stress in plants: Recent insights and trends. Stress Biol..

[110] Shi Y.T., Ding Y.L., Yang S.H. (2018). Molecular regulation of CBF signaling in cold acclimation. Trends Plant Sci..

[111] Wang H., Yin C., Zhang G., Yang M., Zhu B., Jiang J., Zeng Z. (2024). Cold-induced deposition of bivalent *H3K4me3-H3K27me3* modification and nucleosome depletion in *Arabidopsis*. Plant J..

[112] Faiver L., Kinscher N.F., Kuhlmann A.B., Xu X.C., Kaufmann K., Schubert D. (2024). Cold stress induces a rapid redistribution of the antagonistic marks *H3K4me3* and *H3K27me3* in *Arabidopsis thaliana*. bioRxiv.

[113] Guo H., Zhou M., Zhang G., He L., Yan C.H., Wan M., Hu J.J., Zeng Z.X. (2023). Development of homozygous tetraploid potato and whole genome doubling-induced the enrichment of *H3K27ac* and potentially enhanced resistance to cold-induced sweetening in tubers. Hortic. Res..

[114] Gómez-Martínez D., Barrero-Gil J., Tranque E., Ruiz M.F., Catalá R., Salinas J. (2023). *SVALKA*-POLYCOMB REPRESSIVE COMPLEX2 module controls *C-REPEAT BINDING FACTOR3* induction during cold acclimation. Plant Physiol..

[115] Ye K., Li H., Ding Y., Shi Y.T., Song C.P., Gong Z.Z., Yang S.H. (2019). BRASSINOSTEROID-INSENSITIVE2 negatively regulates the stability of transcription factor *ICE1* in response to cold stress in *Arabidopsis*. Plant Cell.

[116] Vyse K., Faivre L., Romich M., Pagter M., Schubert D., Hincha D.K., Zuther E. (2020). Transcriptional and post-transcriptional regulation and transcriptional memory of chromatin regulators in response to low temperature. Front. Plant Sci..

[117] Di Q., Zhou M., Li Y., Yan Y., He C.X., Wang J., Wang X.Q., Yu X.C., Sun M.T. (2024). *RESPIRATORY BURST OXIDASE HOMOLOG 5.1* regulates *H3K4me3* deposition and transcription after cold priming in cucumber. Plant Physiol..

[118] Gao Z., Li Y., Ou Y., Zeng X.L., Li R.J., He Y.H. (2023). A pair of readers of bivalent chromatin mediate formation of Polycomb-based “memory of cold” in plants. Mol. Cell.

[119] Gupta S., Kaur R., Upadhyay A., Chauhan A., Tripathi V. (2024). Unveiling the secrets of abiotic stress tolerance in plants through molecular and hormonal insights. 3 Biotech.

[120] Li J.Y., Wei J.H., Song Y.T., Chen N., Ni B.B., Zhang J.G., He C.Y. (2023). Histone H3K9 acetylation modulates gene expression of key enzymes in the flavonoid and abscisic acid pathways and enhances drought resistance of sea buckthorn. Physiol. Plant..

[121] Li W., Deng M., Wang S., Wang C., Guo M., Song Y., Guo J., Yan J., Ma F., Guan Q. (2023). Histone deacetylase 6 interaction with abscisic acid-insensitive 5 decreases apple drought tolerance. Plant Physiol..

[122] Rajput P., Agarwal P., Agarwal P.K. (2024). A sesamum indicum *SiMYB77* transcription factor enhances drought and salt tolerance in transgenic tobacco via maintaining higher osmolytes and ROS homeostasis. J. Plant Growth Regul..

[123] Han B., Xu W., Ahmed N., Yu A., Wang Z., Liu A. (2020). Changes and associations of genomic transcription and histone methylation with salt stress in castor bean. Plant Cell Physiol..

[124] Basang Y.Z., Zha S., Mu W., Yu M.Z., Wang Y.L., Yuan H.J., Xu Q.J. (2021). Whole-genome analysis of the trimethylation of histone H3 lysine 4 and lysine 27 in two contrasting Tibetan hulless genotypes under salinity stress. Acta Physiol. Plant..

[125] Xu Q.J., Huang S.M., Guo G.G., Yang C.B., Wang M., Zeng X.Q., Wang Y.L. (2022). Inferring regulatory element landscapes and gene regulatory networks from integrated analysis in eight hulless barley varieties under abiotic stress. BMC Genom..

[126] Tian W., Liu T., Chen H., Ma A.J., Wang G.Q., Zhang Y.J., Zhang B. (2025). Integrative analysis of physiological properties and transcriptome reveals the mechanism of salt tolerance in Oat (*Avena sativa* L.). J. Plant Growth Regul..

[127] Zhou X.R., Wang M.M., Yang L., Wang W.P., Zhang Y.H., Liu L.B., Chai J.K., Liu H., Zhao G.Q. (2024). Comparative physiological and transcriptomic analyses of Oat (*Avena sativa*) seedlings under salt stress reveal salt tolerance mechanisms. Plants.

[128] Stadnik B., Tobiasz-Salach R., Mazurek M. (2022). Effect of silicon on Oat salinity tolerance: Analysis of the epigenetic and physiological response of plants. Agriculture.

[129] Shen Y., Chi Y.H., Lu S., Lu H.J., Shi L. (2022). Involvement of JMJ15 in the dynamic change of genome-wide *H3K4me3* in response to salt stress. Front. Plant Sci..

[130] Zhang D., Guo W., Wang T., Wang Y.F., Le L., Xu F., Wu Y., Wuriyanghan H., Sung Z.R., Pu L. (2022). RNA 5-Methylcytosine modification regulates vegetative development associated with H3K27 trimethylation in *Arabidopsis*. Adv. Sci..

[131] Yuan Y.H., Li J., Ma H.C., Yang Q.H., Liu C.J., Feng B.L. (2020). Salt-tolerant broomcorn millet (*Panicum miliaceum* L.) resists salt stress via modulation of cell wall biosynthesis and Na^+^ balance. Land Degrad. Dev..

[132] Lloyd J.P.B., Lister R. (2021). Epigenome plasticity in plants. Nat. Rev. Genet..

[133] Xu Z.S., Chen X.J., Lu X.P., Zhao Y.M., Liu J.H. (2021). Integrative analysis of transcriptome and metabolome reveal mechanism of tolerance to salt stress in oat (*Avena sativa* L.). Plant Physiol. Biochem..

[134] Gao Y., Wang T., Zhao C. (2024). Advances in epigenetic studies of plant cadmium stress. Front. Plant Sci..

[135] Faizan M., Alam P., Hussain A., Karabulut F., Tonny S.H., Cheng S.H., Yusuf M., Adil M.F., Sehar S., Alomrani S.O. (2024). Phytochelatins: Key regulator against heavy metal toxicity in plants. Plant Stress.

[136] Xiao Q.T., Wang Y.J., Lü Q.X., Wen H.H., Han B., Chen S., Zheng X.Y., Lin R.Y. (2020). Responses of glutathione and phytochelatins biosynthesis in a cadmium accumulator of *Perilla frutescens* (L.) britt. under cadmium contaminated conditions. Ecotoxicol. Environ. Saf..

[137] Zheng Y., Li M., Liu B., Qin Y., Li J., Pan Y., Zhang X. (2022). Effects of phytochelatin-like gene on the resistance and enrichment of cd2+ in tobacco. Int. J. Mol. Sci..

[138] Gallo-Franco J.J., Sosa C.C., Ghneim-Herrera T., Quimbaya M. (2020). Epigenetic control of plant response to heavy metal stress: A new view on aluminum tolerance. Front. Plant Sci..

[139] Yang T.Y., Liu Z.S., Pan X.J., Wei W.Y., An C.T., Li L., Wang Y.H., Liao W.B., Wang C.L. (2025). Effect of histone acetylation on plant resistance to salt stress. Plant Soil.

[140] Lu Y., Xu Q., Liu Y., Yu Y., Cheng Z.Y., Zhao Y., Zhou D.X. (2018). Dynamics and functional interplay of histone lysine butyrylation, crotonylation, and acetylation in rice under starvation and submergence. Genome Biol..

[141] Zhao G., Zhao H.Y., Hou X.T., Wang J., Cheng P.F., Xu S., Cui W.T., Shen W.B. (2022). An unexpected discovery toward argon-rich water amelioration of cadmium toxicity in *Medicago sativa* L.. Sci. Total Environ..

[142] Ma Y., Jie H., Zhao L., He P., Lv X., Xu Y., Zhang Y., Xing H., Jie Y. (2024). *BnXTH1* regulates cadmium tolerance by modulating vacuolar compartmentalization and the cadmium binding capacity of cell walls in ramie (*Boehmeria nivea*). J. Hazard. Mater..

[143] Wang C., Wang D., Wang H., Zhao X., You Y., Huang J., Xing M. (2026). HDAC-mediated non-histone deacetylation as a central regulatory network integrating crop growth and stress adaptation. Plant Cell Rep..

[144] Lim J., Lim C.W., Lee S.C. (2022). Core components of abscisic acid signaling and their post-translational modification. Front. Plant Sci..

[145] Liu H.T., Tang X., Zhang N., Li S.G., Si H.J. (2023). Role of bZIP transcription factors in plant salt stress. Int. J. Mol. Sci..

[146] Wang X., Liu W.C., Zeng X.W., Yan S., Qiu Y.M., Wang J.B., Huang X., Yuan H.M. (2021). HbSnRK2.6 functions in ABA-regulated cold stress response by promoting HbICE2 transcriptional activity in *Hevea brasiliensis*. Int. J. Mol. Sci..

[147] Benderradji L., Saibi W., Brini F. (2021). Role of ABA in overcoming environmental stress: Sensing, signaling and crosstalk. Ann. Agric. Crop Sci..

[148] Yu Y., Zhao F., Yue Y., Zhao Y., Zhou D.X. (2024). Lysine acetylation of histone acetyltransferase adaptor protein ADA2 is a mechanism of metabolic control of chromatin modification in plants. Nat. Plants.

[149] Satyakam, Zinta G., Singh R.K., Kumar R. (2022). Cold adaptation strategies in plants—An emerging role of epigenetics and antifreeze proteins to engineer cold resilient plants. Front. Genet..

[150] Park J., Jung J.H. (2024). Revalidation of the *ICE1–CBF* regulatory model in *Arabidopsis* cold stress response. J. Plant Biol..

[151] Zhang X., Yu J., Qu G., Chen S. (2024). The cold-responsive C-repeat binding factors in *Betula platyphylla* Suk. positively regulate cold tolerance. Plant Sci..

[152] Kim J., Jeong R.E., Sung S. (2026). Chromatin remodeling at the C-repeat binding factor cluster controls growth retardation at low ambient temperature. Plant Cell.

[153] Wang Y., Wang J., Sarwar R., Zhang W., Geng R., Zhu K.-M., Tan X.-L. (2024). Research progress on the physiological response and molecular mechanism of cold response in plants. Front. Plant Sci..

[154] Zhao W.Y., Xu Y., Wang Y.F., Gao D., King J., Xu Y.J., Liang F.S. (2021). Investigating crosstalk between H3K27 acetylation and H3K4 trimethylation in CRISPR/dCas-based epigenome editing and gene activation. Sci. Rep..

[155] Bao L., Wang W., Li M., Liu J., Liu J., Alifu G., Wang D., Liang X., Mao T., Zhai Y. (2025). Reactive oxygen species-post translational modifications-central carbon metabolism regulatory loop: Coordination of redox homeostasis and carbon flux allocation in plants under abiotic stress. Front. Plant Sci..

[156] Fedoreyeva L.I. (2024). ROS as signaling molecules to initiate the process of plant acclimatization to abiotic stress. Int. J. Mol. Sci..

[157] Huang H., Ullah F., Zhou D.X., Yi M., Zhao Y. (2019). Mechanisms of ROS regulation of plant development and stress responses. Front. Plant Sci..

[158] Mittler R., Zandalinas S.I., Fichman Y., Van Breusegem F. (2022). Reactive oxygen species signalling in plant stress responses. Nat. Rev. Mol. Cell Biol..

[159] Rosenfeld M.A., Yurina L.V., Gavrilina E.S., Vasilyeva A.D. (2024). Post-translational oxidative modifications of hemostasis proteins: Structure, function, and regulation. Biochemistry.

[160] Kim J.M., Sasaki T., Ueda M., Sako K., Seki M. (2015). Chromatin changes in response to drought, salinity, heat, and cold stresses in plants. Front. Plant Sci..

[161] Kim J.H. (2021). Multifaceted Chromatin Structure and Transcription Changes in Plant Stress Response. Int. J. Mol. Sci..

[162] Sintaha M. (2025). Molecular mechanisms of plant stress memory: Roles of non-coding RNAs and alternative splicing. Plants.

[163] He Y.H., Li Z.C. (2018). Epigenetic environmental memories in plants: Establishment, maintenance, and reprogramming. Trends Genet..

[164] Aswathi K.P.R., Ul-Allah S., Puthur J.T., Siddique K.H.M., Frei M., Farooq M. (2025). The plant mind: Unraveling abiotic stress priming, memory, and adaptation. Physiol. Plant..

[165] Song S., Wang Y., Wang J., Liu Y., Zhang X., Yang A., Li F. (2024). Low *H3K27me3* deposition at *CYP82E4* determines the nicotinic conversion rate in *Nicotiana tabacum*. Plant Physiol. Biochem..

[166] Rehman S., Ahmad Z., Ramakrishnan M., Kalendar R., Zhuge Q. (2023). Regulation of plant epigenetic memory in response to cold and heat stress: Towards climate resilient agriculture. Funct. Integr. Genom..

[167] Hereme R., Galleguillos C., Morales-Navarro S., Molina-Montenegro M.A. (2021). What if the cold days return? Epigenetic mechanisms in plants to cold tolerance. Planta.

[168] Shi L., Cui X.Y., Shen Y. (2024). The roles of histone methylation in the regulation of abiotic stress responses in plants. Plant Stress.

[169] Yamaguchi N., Matsubara S., Yoshimizu K., Seki M., Hamada K., Kamitani M., Kurita Y., Nomura Y., Nagashima K., Inagaki S. (2021). *H3K27me3* demethylases alter HSP22 and HSP17.6C expression in response to recurring heat in *Arabidopsis*. Nat. Commun..

[170] Larran A.S., Pajoro A., Qüesta J.I. (2023). Is winter coming? Impact of the changing climate on plant responses to cold temperature. Plant Cell Environ..

[171] Shriti S., Bhar A., Roy A. (2024). Unveiling the role of epigenetic mechanisms and redox signaling in alleviating multiple abiotic stress in plants. Front. Plant Sci..

[172] Di Q., Liu L., Xie K., Yan Y., Zhou M., He C., Li Y., Yu X., Wang J., Sun M. (2025). Genome-wide analysis of *H3K27me3* in cucumber during recovery after cold stress priming. Plant Physiol. Biochem..

[173] Zhu Z., Li Q., Gichuki D.K., Hou Y., Liu Y., Zhou H., Xu C., Fang L., Gong L., Zheng B. (2023). Genome-wide profiling of histone H3 lysine 27 trimethylation and its modification in response to chilling stress in grapevine leaves. Hortic. Plant J..

[174] Rahman A., Yadav N.S., Byeon B., Ilnytskyy Y., Kovalchuk I. (2024). Genomic and epigenomic changes in the progeny of cold-stressed *Arabidopsis thaliana* Plants. Int. J. Mol. Sci..

[175] Guo J., Wang H., Liu S., Wang Y., Liu F., Li X. (2022). Parental drought priming enhances tolerance to low temperature in wheat (*Triticum aestivum*) offspring. Funct. Plant Biol..

[176] Jogam P., Sandhya D., Alok A., Peddaboina V., Allini V.R., Zhang B. (2022). A review on CRISPR/Cas-based epigenetic regulation in plants. Int. J. Biol. Macromol..

[177] Junaid M.D., Chaudhry U.K., Şanlı B.A., Gökçe A.F., Öztürk Z.N. (2024). A review of the potential involvement of small RNAs in transgenerational abiotic stress memory in plants. Funct. Integr. Genom..

[178] He S.B., Zhang P.H., Wang Y.H., Zheng R., Li Y.Q., Cheng H., Lv D., Sun Y.F., Miao C. (2024). Transcriptional patterns and histone modification signatures reveal dehydration memory behaviour in seedlings of *Gossypium hirsutum*. Environ. Exp. Bot..

[179] Kumar S., Seem K., Mohapatra T. (2023). Biochemical and epigenetic modulations under drought: Remembering the stress tolerance mechanism in rice. Life.

[180] Liu H.P., Able A.J., Able J.A. (2022). Priming crops for the future: Rewiring stress memory. Trends Plant Sci..

[181] Zhu D., Wen Y., Yao W., Zheng H., Zhou S., Zhang Q., Qu L.-J., Chen X., Wu Z. (2023). Distinct chromatin signatures in the Arabidopsis male gametophyte. Nat. Genet..

[182] Gahlaut V., Jaiswal V. (2026). Transposable elements: Mediators of epigenetic inheritance in plants. Trends Plant Sci..

[183] Corrêa R.L., Kutnjak D., Ambrós S., Bustos M., Elena S.F. (2024). Identification of epigenetically regulated genes involved in plant-virus interaction and their role in virus-triggered induced resistance. BMC Plant Biol..

[184] Liu B.B., Zhao M.X. (2023). How transposable elements are recognized and epigenetically silenced in plants?. Curr. Opin. Plant Biol..

[185] Chen Y., Hung F.Y., Sugimoto K. (2023). Epigenomic reprogramming in plant regeneration: Locate before you modify. Curr. Opin. Plant Biol..

[186] Lövkvist C., Mikulski P., Reeck S., Hartley M., Dean C., Howard M. (2021). Hybrid protein assembly-histone modification mechanism for prc2-based epigenetic switching and memory. eLife.

[187] Cheong J.J. (2023). Transcription control mechanisms for plant stress responses. Int. J. Mol. Sci..

[188] Li Y., Chen M., Khan A.H., Ma Y., He X., Yang J., Zhang R., Ma H., Zuo C., Li Y. (2023). Histone h3 lysine 27 trimethylation suppresses jasmonate biosynthesis and signaling to affect male fertility under high temperature in cotton. Plant Commun..

[189] Malik S., Zhao D. (2022). Epigenetic regulation of heat stress in plant male reproduction. Front. Plant Sci..

[190] Adane M., Alamnie G. (2024). CRISPR/cas9 mediated genome editing for crop improvement against abiotic stresses: Current trends and prospects. Funct. Integr. Genom..

[191] Rao Y., Yang X., Pan C., Wang C., Wang K. (2022). Advance of clustered regularly interspaced short palindromic repeats-cas9 system and its application in crop improvement. Front. Plant Sci..

[192] Joshi A., Yang S.Y., Song H.G., Min J., Lee J.H. (2023). Genetic databases and gene editing tools for enhancing crop resistance against abiotic stress. Biology.

[193] Mohamed H.I., Khan A., Basit A. (2024). Crispr-cas9 system mediated genome editing technology: An ultimate tool to enhance abiotic stress in crop plants. J. Soil Sci. Plant Nutr..

[194] Kumar M., Prusty M.R., Pandey M.K., Singh P.K., Bohra A., Guo B., Varshney R.K. (2023). Application of crispr/cas9-mediated gene editing for abiotic stress management in crop plants. Front. Plant Sci..

[195] Roca Paixão J.F., Gillet F.X., Ribeiro T.P., Bournaud C., Lourenço-Tessutti I.T., Noriega D.D., Paes de Melo B.P.M., de Almeida-Engler J.A.E., Grossi-de-Sa M.F.G. (2019). Improved drought stress tolerance in Arabidopsis by crispr/dcas9 fusion with a histone acetyltransferase. Sci. Rep..

[196] Lee J.E., Neumann M., Duro D.I., Schmid M. (2019). Crispr-based tools for targeted transcriptional and epigenetic regulation in plants. PLoS ONE.

